# Critical review of the TransCelerate Template for clinical study reports (CSRs) and publication of Version 2 of the CORE Reference (*C*larity and *O*penness in *R*eporting: *E*3-based) Terminology Table

**DOI:** 10.1186/s41073-019-0075-5

**Published:** 2019-08-05

**Authors:** Samina Hamilton, Aaron B. Bernstein, Graham Blakey, Vivien Fagan, Tracy Farrow, Debbie Jordan, Walther Seiler, Art Gertel, on behalf of the Budapest Working Group [BWG]

**Affiliations:** 1 European Medical Writers Association, Chester House, 68 Chestergate, Macclesfield, Cheshire SK11 6DY UK; 2 American Medical Writers Association, 30 W. Gude Drive, Ste. 525, Rockville, MD 20850-4347 USA; 3 Sam Hamilton Medical Writing Services Limited, Newcastle Upon Tyne, UK; 4 Aaron Bernstein Consulting, LLC, Millburn, NJ USA; 5 Consult2Deliver, BioCity, Pennyfoot Street, Nottingham, NG1 1GF UK; 6 IQVIA, The Alba Campus, Rosebank, Livingston, West Lothian EH54 7EG UK; 7 PPD, Granta Park, Great Abingdon, Cambridge, CB21 6GQ UK; 8 Debbie Jordan Limited, Hook, Hampshire UK; 9 Bayer AG, 13342 Berlin, Germany; 10 MedSciCom, LLC, Lebanon, NJ USA

**Keywords:** Research Report, Guideline, Patient Data Privacy, Randomized Controlled Trial, Disclosure, Information Dissemination, Data Reporting

## Abstract

**Background:**

CORE (*C*larity and *O*penness in *R*eporting: *E*3-based) Reference (released May 2016 by the European Medical Writers Association [EMWA] and the American Medical Writers Association [AMWA]) is a complete and authoritative open-access user’s guide to support the authoring of clinical study reports (CSRs) for current industry-standard-design interventional studies. CORE Reference is a content guidance resource and is not a CSR Template.

TransCelerate Biopharma Inc., an alliance of biopharmaceutical companies, released a CSR Template in November 2018 and recognised CORE Reference as one of two principal sources used in its development.

**Methods:**

The regulatory medical writing and statistical professionals who developed CORE Reference conducted a critical review of the TransCelerate CSR Template. We summarise our major findings and recommendations in this communication. We also re-examined and edited the Version 1 CORE Reference Terminology Table that we first published in 2016, and we present this as Version 2 in this communication.

**Results:**

Our major critical review findings indicate that opportunities remain to refine the CSR Template structure and instructional text, enhance content clarity, add web links to referenced guidance documents, improve transparency to support the broad readership of CSRs, and develop supporting resources.

The CORE Reference ‘Terminology Table’ Version 2 includes estimand as a defined term and an adaptation of the original ‘worked study example’ to incorporate the recently evolved concept of ‘estimands’.

**Conclusions:**

As TransCelerate’s CSR Template represents an important milestone in authoring CSRs, we offer CSR authors advice and recommendations on its use, similarities, and differences with CORE Reference and advise them to consider shared interpretations between the two.

**Registration:**

CORE Reference is registered with the EQUATOR Network. The TransCelerate CSR Template is not registered with any external organisation to the knowledge of the authors of this paper.

**Electronic supplementary material:**

The online version of this article (10.1186/s41073-019-0075-5) contains supplementary material, which is available to authorized users.

## Background

Developed by the Budapest Working Group (BWG), comprising European Medical Writers Association (EMWA) and American Medical Writers Association (AMWA) members in a 2-year pro bono effort, CORE (*C*larity and *O*penness in *R*eporting: *E*3-based) Reference [1] published in May 2016, and registered with the Equator Network [2], is a complete and authoritative open-access user’s guide to support the authoring of CSRs for interventional studies [3]. It is complete because it includes all the necessary guidance components for reporting complex modern-design clinical studies, whilst maximising both personal data protections and data utility. It is authoritative because International Council for Harmonisation (ICH) guidance documents, as well as the European Union (EU), i.e. European Medicines Agency (EMA), and the USA, i.e. Food and Drug Administration (FDA) regional guidelines, were accessed, analysed, and interpreted. As a number of important stakeholders were involved in its development, they were able to provide insights with regard to industry best practices. As per the underlying ICH E3 document, CORE Reference is a reference tool companion to ICH E3 and not a template. It offers sectional content suggestions but does not mandate a particular CSR structure. The content suggestions are arranged in sections, which are mapped to the principal regulatory guidance, ICH E3 [4], using the CORE Reference mapping tool. This allows users to contextualise CORE Reference within ICH E3.

In the 3 years since CORE Reference was launched, the associated website [5] has evolved into a living and multi-dimensional educational resource for CSR authors and reviewers. The PDFs of CORE Reference and the ICH E3-CORE Reference mapping tool are available to download [1]; regulatory and disclosure news updates are sent in real time to those subscribing for email updates [6]; and these are periodically summarised and posted on the website [7].

Additionally, since CORE Reference was first published, the global transparency and disclosure landscape has evolved. Mandatory public disclosure of CSRs submitted as part of a regulatory dossier to the EMA has been suspended since August 2018 with no planned date for lifting of the suspension [8]. When the suspension is lifted, clinical data publication will be subject to new implementation guidance (for Policy 0070 Version 1.4, dated 15 October 2018) that takes into account lessons learned from the EMA, sponsors, and the pharmaceutical industry over 2 years of submissions [9]. In January 2018, the FDA launched a pilot programme ‘…to evaluate whether disclosing certain information included within CSRs following approval of a New Drug Application improves public access to drug approval information’, and subsequently issued a FAQ document in May 2018 [10]. In June 2019, the FDA sought public feedback on the pilot, and additionally on a new integrated review process and template, declaring the Agency is considering ‘…whether to focus its efforts to better communicate the basis for drug approvals on the development of new integrated review documents, rather than on the release of CSRs’ [10]. The only CSR publicly posted from the FDA pilot is that of the Erleada® pivotal trial [11]. Health Canada has issued guidance on the public release of clinical information, which largely aligns with EMA guidance [12]. Japan, Australia, and Germany also have requirements for the disclosure of product submission data [13]. Although the disclosure of clinical trial data is currently in a holding phase across multiple regions, it will resume. In the meantime, CSR authors are advised to maintain current awareness of global requirements for data disclosure [14] and to continue writing CSRs in a proactively anonymised fashion that maximises data utility, as mandated in Policy 0070 current implementation guidance [9] and as described in CORE Reference [1].

TransCelerate is an alliance among some of the world’s prominent biopharmaceutical organisations with the tagline ‘accelerating the development of new medicines’. TransCelerate provides solutions that ‘…are developed collaboratively and can be voluntarily adopted by stakeholders in the clinical research ecosystem’ [15]. In November 2018, TransCelerate released its first version of a CSR Template. This CSR Template and associated resources, including a template for statistical analysis plans (SAPs), are located on the TransCelerate website under the ‘Common Protocol Template’ resources tab [16]. A tool showing the TransCelerate CSR Template mapped to ICH E3 and CORE Reference is additionally provided [17].

CORE Reference is a broadly recognised resource as evidenced by 20,000+ downloads by June 2019, and adoption and use testimonials originating from Europe, the USA, Asia Pacific, and Africa [18]. TransCelerate recognises CORE Reference as one of two principal sources used in its CSR Template development. The other is ICH E3 [4]. A template created using these two authoritative references could represent a highly complementary ‘next step’ by the global pharmaceutical industry. Guidance documents maximise their utility if explanations, together with supportive sources, are included, and this approach also has educational value for the ever-growing pool of industry professionals responsible for contribution to, or preparation of, CSRs. The value to reviewers should not be overlooked given the commonality of presentation philosophy. Reviewers will be able to locate categorical information more easily and will more likely see compliance with ICH E3.

The aim of this paper was for the Budapest Working Group (BWG)—the developer of CORE Reference—to conduct a critical review of a CSR Template that was developed by TransCelerate. TransCelerate used CORE Reference to develop their CSR Template but did not involve the BWG in its development. We, the BWG, have conducted a thorough critical review and analysis of the TransCelerate CSR Template and summarise our major findings here to expand on our initial observations on the TransCelerate CSR Template, which we published in a press release in December 2018 [19]. We also took this opportunity to review and update the original Version 1 CORE Reference ‘Terminology Table’ and present Version 2, which includes estimand as a defined term and an adaptation of the original ‘worked study example’ to incorporate the recently evolved concept of ‘estimands’.

## Methods

### Critical review of the TransCelerate CSR Template and resulting outputs

Over the period 11 January to 28 March 2019, the BWG discussed similarities and differences between the TransCelerate CSR Template and CORE Reference. The BWG’s comments on the TransCelerate CSR Template were collated and proof-checked, and the resulting unabridged critical review comments are provided in Additional file 1. We classified a proportion of the review comments as major findings. In Table 1, we summarise these, and we make recommendations on enhancements that may be applied to the TransCelerate CSR Template with respect to each major finding. Finally, this manuscript was drafted and underwent review by the BWG.

**Table 1:** The BWG’s major critical review findings on and recommendations for enhancing TransCelerate’s CSR Template

Additional file 1 text location	Major critical review finding	Recommendation for CSR Template enhancement
About this template/page 2	The red instructional text relating to the Core Backbone Headings is somewhat inflexible with respect to Level 2 headings order: ‘Level 2 and lower headings can be deleted/added/modified, as needed, however do not rearrange or reorder sections’	Allow rearrangement or reordering of Level 2 and lower hierarchy headings
Red instructional text/page 4 and section 7/page 44	TransCelerate CSR Template red instructional text states: ‘For recommendations related to using this template given the disclosure requirements, please refer to the best practices for disclosure in the implementation toolkit available on TransCelerate’s website’ and ‘Reference ICH E3 and CORE (*C*larity and *O*penness in *R*eporting: *E*3-based) for guidance on the naming/numbering of tables/listing/figures and appendices’. Links to these resources are missing.	Add these direct links in the instructional text to improve Template utility: • www.core-reference.org (the website from which CORE Reference may be downloaded) • https://www.ich.org/products/guidelines/efficacy/article/efficacy-guidelines.html (the ICH efficacy guidelines webpage from which ICH E3 may be downloaded) • https://transceleratebiopharmainc.com/initiatives/clinical-data-transparency (TransCelerate’s Clinical Data Transparency Page)
Title page/page 6	Omission	Include an ICH-GCP compliance statement on the title page
Synopsis/page 8	Omission	Include ‘Background and rationale for the study’ in the synopsis
Synopsis, example Tables 1 and 2/page 9	Example tables should be relocated and edited	Position example Tables 1 and 2—that summarise objective, endpoints/estimands—earlier in the synopsis before the methods, so that the methods may be more easily contextualised. Delete the ‘Results’ columns from these example tables as this effectively places ‘Results’ into the synopsis ‘Methods’
Ethics: ‘Ethical Conduct of the Study’/page 20	Inappropriate listing of local laws	Avoid listing local laws that underpin the Informed Consent Form, and keep the example wording more general, e.g., ‘…local laws and applicable data privacy regulations’
Ethics: ‘Participant information and consent’/page 20	Omission	In the interests of transparency, state the location of the master Patient Information Sheet and Informed Consent Form (i.e. in the Trial Master File)
Section 3.1/pages 23 and 24; Section 3.3.1/page 25; Section 3.4.2/page 27; Section 3.5.1/page 28; Section 3.7/page 30	Overuse of cross-referencing the protocol in various places in the CSR Template	Cross-referencing to external documents, such as the protocol, should be done judiciously. Comprehension of the CSR as a stand-alone document should not be negatively impacted because of extensive streamlining. Also consider the likelihood that a protocol cross-link would typically navigate to the start of the protocol and not to the relevant section therein. Where particular topics are not well-described in the protocol, extensive cross-referencing would reinforce poor descriptions/explanations when the CSR should allow an opportunity to clarify those topics
Section 3.6 ‘Data Quality Assurance’/page 29	Omission	Add a sub section under Section 3.6 to describe the quality management approach and summarise key issues
Example Figure X/page 33	General omission	Include the study number in the header of all in-text tables and in-text figures. This directly supports the regulatory authorities, as communicated to the BWG by the EMA, and aligns with the CORE Reference approach
Example Figure X/page 33	Multiple computer hardware and software packages and combinations exist. Therefore, editable figures may not always appear on screen in the intended format	Provide PDF file images of figures, such as ‘Figure X’, as a supplementary asset to support re-creation of (editable) figures by the end user if needed
Section 4.2 ‘Protocol Deviations’/page 34	Omission	Add a separate (sub)section for any audit findings or centre-specific violations, e.g., if centres were identified with potential misconduct, identify those centre IDs only in Section 4.2 Protocol Deviations. In other parts of the CSR, suggest cross referencing this section to avoid repeated redaction
Section 4.6.3 ‘Measurement of Compliance’/page 35	Lack of clarity in the section title	Suggest editing the Section 4.6.3 title to ‘Compliance with study intervention’, or a similar amended title, could make a clearer distinction between this intended sectional content and the intended sectional content for the Protocol Deviations section
Section 5.2/page 37	General omission of advisory text on proactive anonymisation and data transparency	Add specific tips to support proactive authoring and data transparency that will minimise redaction in the publicly-disclosed version of the CSR. These may be used as instructional text: • Subsections with subheadings are recommended for individual subject information (to ease redaction) • Subject IDs (if needed) should be listed directly adjacent to other personal information, e.g., subject’s age or sex (to ease redaction) • Avoid using sex-specific language like ‘he/she, his/her’, and replace with ‘the subject(s)’. Similarly, use ‘subject’s spouse/partner’ instead of ‘subject’s husband/wife’ • Avoid including summaries of narratives (mini-narratives) in the body of the CSR
		• Avoid use of investigator verbatim text that could include clues to the identity of the subject • Prefix each subject ID with ‘#’. This makes subject IDs easily searchable (to ease redaction)
Section 5.2.1 ‘Adverse Events’/page 38	Multiple omissions	Include more extensive instructional text, as well as a comprehensive set of Adverse Event in-text summary tables, to support appropriate Adverse Event reporting. In addition, there are multiple opportunities to enhance the Adverse Event reporting section, as detailed in the BWG comments in Appendix 1
Section 5.2.2 ‘Clinical Laboratory Evaluation’/page 40	Omission	Include more extensive instructional text to support appropriate reporting, as detailed in the BWG comments in Appendix 1
Section 5.2.3 ‘Other Safety Evaluations’/page 41	Omission	Include more extensive instructional text to support appropriate reporting, particularly with regard for medical devices, and as detailed in the BWG comments in Appendix 1
Section 5.3 ‘Pharmacokinetics through Section 5.6 Biomarkers’/page 42	Multiple omissions	Include more extensive instructional text. Guidance on the points for inclusion are detailed in the BWG comments in Appendix 1
Section 5.10 ‘Summary of Evaluation of Response to Study Intervention’/page 43	Inappropriate instruction	Make this a mandatory, not an optional section
General page 44	Omission	Include a Discussion section to the CSR Template. This is currently missing
Page 45	Omission	Include instructional text around the presentation of the in-text tables and figures as well as the content of the appendices
General	Omission	The CSR Template should undergo quality control, including review of all internal cross-links

The BWG’s major findings included in Additional file 1 are represented in Table 1. For each Table 1 major finding, we make specific recommendations for enhancing the TransCelerate CSR Template. To view all critical review comments (major and minor) in situ and, therefore, in context and with supporting detail, view Additional file 1

The original CORE Reference Terminology Table (page 32 of Version 1.0 CORE Reference [1]) was updated to include estimands in the resulting Version 2.0 Terminology Table. The Version 2.0 CORE Reference Terminology Table is presented in Table 2. Despite the suggestion that (predominantly) later patient-focused safety and efficacy studies lend themselves to having estimands, we also consider their applicability to early-phase development.

**Table 2:**
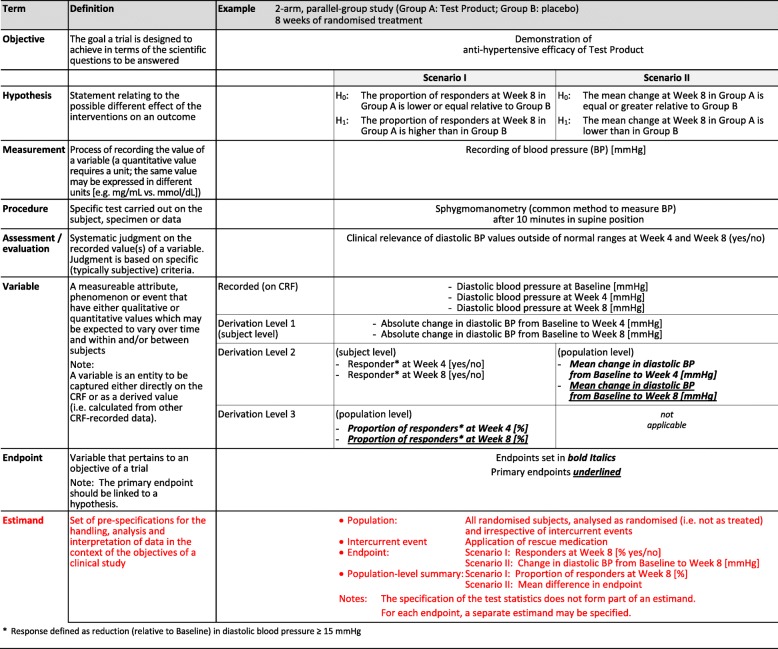
Version 2.0 of the CORE Reference Terminology Table

Table 2 is Version 2.0 of the original CORE Reference Terminology Table, first published integral to CORE Reference (Version 1.0 dated 03 May 2016). The red text represents the updated text on estimands. This updated educational resource is also posted at www.core-reference.org

^*^Response defined as reduction (relative to baseline) in diastolic blood pressure ≥ 15 mmHg

## Results

### Results of the review of the TransCelerate CSR Template

For each major finding in Table 1, we make suggestions for enhancements to the TransCelerate CSR Template. These can be viewed as opportunities to refine the CSR Template structure and instructional text, enhance content clarity, add web links to referenced guidance documents, improve transparency to support the broad readership of CSRs, and develop supporting resources. These are our key recommendations for consideration in the further development of the TransCelerate CSR Template or for sponsor adaptations thereof. To view all of the review team comments in situ and, therefore, in context and with supporting detail, we recommend viewing Additional file 1. 

### Version 2.0 of the CORE Reference Terminology Table

Following the 2016 publication of CORE Reference, the concept of ‘estimand’—which was mentioned in CORE Reference as a future expected area of interest—has matured and is now better understood. The TransCelerate CSR Template takes estimand into consideration. We believe that the update of the CORE Reference Terminology Table to include estimand is now timely.

‘Estimand’, unlike the other terms already included in the Terminology Table, encompasses several different aspects. The proposed definition of the term ‘estimand’ follows the requirements for definitions previously applied in the Terminology Table. For ease of comprehension, the example chosen for estimand extrapolates the examples for the already existing terms.

The updated Version 2.0 of the CORE Reference Terminology Table is presented in Table 2.

## Discussion

Although independently developed, the CORE Reference and TransCelerate CSR Template both serve as resources that can be voluntarily adopted by stakeholders in the clinical research ecosystem, and help achieve better compliance with global standards for reporting clinical study data, analysis, and interpretation.

The BWG views the TransCelerate CSR Template as a valuable addition to the available resources for CSR authors and reviewers. Although CORE Reference serves a different purpose, there are synergies between the two, as shown in the Additional file 1 comments. There are, however, differences, which broadly fit into the themes discussed below, and include transparency and public disclosure, structural flexibility, section-to-section continuity, sectional numbering, and instructional text completeness and clarity.

Transparency and collaboration are necessary in today’s clinical research environment. Cooperation between resource developers can provide a coherent message that directs towards a useable set of global standards. Direct web links to referenced materials and resources increase resource accessibility and provide valuable context and transparency. The CORE Reference website includes web links to the TransCelerate CSR Template resources under ‘Third-party Publications and Presentations’ [20]. We encourage reciprocation in the TransCelerate CSR Template of web links, not only to CORE Reference [5] but also to other guidelines directly referenced but not currently linked in the TransCelerate CSR Template. These include the ICH efficacy guidelines webpage [21] where ICH E3 may be downloaded from and TransCelerate’s Clinical Data Transparency page [22]. It should also be noted that CORE Reference is the only resource that we know of that identifies all current places in an ICH E3-compliant CSR where disclosure considerations apply. This is particularly important because CSRs are now publicly disclosed by the medicine regulators in the EU [8, 9] and Canada [12], and special care is therefore needed when writing CSRs to balance text and data presentations so that on one hand, the identity of individuals is protected, and on the other hand, data utility is optimised to support medicine regulators in unimpeded assessment of license applications [9]. Direct links in the CSR Template to resources that support the writer in this endeavour are, therefore, essential. Further, the TransCelerate CSR Template, the Statistical Analysis Plan (SAP) Template—intended for use by biostatisticians in authoring the SAP (a document that details the statistical methodology typically summarised in a CSR)—and the mapping tool could be relocated on the TransCelerate website, perhaps to a new archive of ‘clinical study reporting assets’ which may help users to find them more easily. Both the CSR and the SAP Templates currently reside under the Common Protocol Template assets. A tool showing the TransCelerate CSR Template, mapped to ICH E3 and CORE Reference, is not co-located with these resources and is difficult to find [17].

A single CSR Template cannot address all study designs without some degree of inherent structural flexibility. There should be the ability to rearrange sections to suit the study design. This is currently lacking due to the TransCelerate CSR Template red instructional text (which is shown in Additional file 1) to not rearrange or reorder Level 1, Level 2, and lower hierarchy headings. Inflexibility in Level 1 headings could reasonably be justified, for example, by regulatory authority reviewer preference, or as a precursor to future Template automations and more sophisticated review tools. The absence of a clear explanation for the required Level 1 heading rigidity could be an impediment to appropriate use of the Template. The inflexibility in Level 2 and lower headings is less easy to justify.

The TransCelerate CSR Template authors recommend the presentation of only primary and secondary endpoints in the synopsis, an optional results summary section, and complete omission of the CSR discussion section in the main Template body. The main body results, summary results, and discussion sections should allow for continuity of results presentation. This is optimal if all endpoints—primary, secondary, and exploratory—are presented in all of these sections. Further, the opportunity for discussion of relevant study-related results must be within the CSR for a given study—to aid comprehension. The absence of such a reporting approach significantly hinders the CSR author, the regulator, and the expanded (public) audience with both authoring and interpreting the document. In CORE Reference page 6, we state ‘…the synopsis should provide a summary of all relevant results (e.g. if there are multiple endpoints, consider limiting to primary and secondary)…’ The flexibility to limit the synopsis presentation to primary and secondary endpoints in CORE Reference was to help achieve consistency in reporting with some clinical trial databases (for example, clinicaltrials.gov and EudraCT) where only primary and secondary endpoints need to be reported. We recognise that inclusion or exclusion of exploratory endpoints in a CSR synopsis may well be based on sponsor preference.

TransCelerate CSR sectional numbering, including those of the CSR appendices, is different from ICH E3 [4] and CORE Reference [1]. Both of these resources proffer content guidance and do not mandate a structure. However, as the ICH E3 numbering system is still frequently referred to in the context of clinical development, TransCelerate should consider aligning their numbering and adapting their mapping tool accordingly.

In the absence of sufficiently detailed TransCelerate CSR Template instructional text, the CSR author would be challenged to develop appropriate sectional content without referring to other guidelines, including CORE Reference. Instructional text as it currently stands in the TransCelerate CSR Template is too lean to support adequate authoring in sections on reporting of adverse events, clinical laboratory evaluation, other safety evaluations (medical devices in particular), pharmacokinetics (PK), pharmacodynamics, biomarkers, and immunogenicity. Further, these sections could be enhanced by the addition of example shell in-text tables. In this predominantly safety- and efficacy-focused CSR Template, there is an opportunity to add value for programmes in early clinical development by enhancing the instructional text that could inform reporting for studies with pharmacological components, e.g. PK and pharmacodynamics. For example, clarification that tabulation of summary PK data and that presentation of summary concentration-time plots (or similar) are appropriate, and that the results should be presented for any PK modelling of the drug concentration-time data, could be beneficial.

CORE Reference had multiple stakeholders involved through its development, including regulatory authority contribution from Health Canada. TransCelerate has not declared any wider stakeholder input—nor has it declared input from any regulatory authority—into the development of their CSR Template. Declaration of stakeholder input reflects the integration of a range of perspectives into any resource’s development and can reasonably be expected to impact its compliance and uptake; therefore, it could be beneficial if TransCelerate added this declaration.

The updated CORE Reference Terminology Table is presented here as Table 2. The concept of estimands as defining quantities that must be estimated from clinical trial data—that take into account post-randomisation events—is well explained by Bridge and Schindler [23]. It is clear that estimands can be readily applied to safety and efficacy studies in patients; further, estimands are expected to be incorporated into SAPs, per the 08 May 2019 ICH E8(R1) draft guidance ‘General Considerations for Clinical Studies’ [24] which is under public consultation at the time of publication of this paper. Early-phase development studies such as first-in-human are exploratory and generally hypothesis-generating. The use of an estimand in these circumstances is less obvious. Nevertheless, it may be possible to choose study endpoints that would inform the estimand in later phase studies, and could provide an estimand-related thread through a medicine’s development. Although not directly applicable to reporting, this consideration may have value at the study design stage.

## Conclusions

This publication will help CSR authors appreciate the distinct values of, and differences between, the TransCelerate CSR Template and CORE Reference. Application of CORE Reference in CSR authoring provides additional instructional value and perspective for users of the TransCelerate CSR Template. CORE Reference maintains its considerable original value as a well-contextualised educational resource containing clarifications and sourcing for each guidance point. TransCelerate’s CSR Template is a unified industry development, built using existing guidance documents, including CORE Reference, and as such is an important milestone. Perhaps a future step could be the collaboration and creation of an integrated ‘tool kit’ for CSR authors.

## Additional file

Additional file 1: The BWG's unabridged critical review comments on TransCelerate's CSR Template. (PDF 488 kb)

## Data Availability

Not applicable.

## Abbreviations

AMWA: American Medical Writers Association
BWG: Budapest Working Group
CORE Reference: Clarity and Openness in Reporting: E3-based Reference
CSR: Clinical study report
EMA: European Medicines Agency
EMWA: European Medical Writers Association
FDA: US Food and Drug Administration
ICH: International Council for Harmonisation
PK: Pharmacokinetic
SAP: Statistical analysis plan
