# Supplementary material for: Critical review of the TransCelerate Template for clinical study reports (CSRs) and publication of Version 2 of the CORE Reference (Clarity and Openness in Reporting: E3-based) Terminology Table
Source: Res Integr Peer Rev. 2019 Aug 5;4:16. doi: 10.1186/s41073-019-0075-5 (PMC6683477; doi:10.1186/s41073-019-0075-5)
Supplement: Supplementary file 1 — The BWG's unabridged critical review comments on TransCelerate's CSR Template. (PDF 488 kb) [file 41073_2019_75_MOESM1_ESM.pdf]

ADDITIONAL FILE 1: Critical Review of the TransCelerate Template for Clinical Study Reports (CSRs) and Publication of Version 2 of the CORE Reference (Clarity and Openness in Reporting: E3-based) Terminology Table.

Hamilton S, Bernstein AB, Blakey G; Fagan V; Farrow T, Jordan D, Seiler W, Gertel A (the Budapest Working Group [BWG]).

- The BWG's unabridged critical review comments on TransCelerate's clinical study report template are provided in the following pages
- Major findings are colour-coded and presented as '[BWG#]: Manuscript Table 1 Major Critical Review Finding'
- Major findings, together with recommendations on relevant enhancements that may be applied to the TransCelerate CSR template in respect of each major finding are presented in Table 1 of the paper
- Minor findings/minor recommendations are not coloured and are presented as '[BWG#]:' in Additional File 1 only.

For ease of navigation, the following table of contents includes links to the beginning of each section:

**About This Template**
**Title Page**
**Synopsis**
**Table of Contents**
**List of Abbreviations and Definition of Terms**
**Ethics**
**1. Introduction**
**2. Study Objectives and [Estimands [and/or] Endpoints**
**3. Investigational Plan**
**4. Study Participants**
**5. Evaluation of Response to Study Intervention**
**6. Conclusions**
**7. References**

Common CSR Template v1.0

About This Template

Disclaimer

This document is a common clinical study report (CSR) template. It contains sections marked as common text or text that may be used across studies with little to no editing if the user chooses to do so. The use of this template is at the discretion of the user. Recommendations for modifications in future releases of the common CSR template can be submitted at any time and will be reviewed on a routine basis.

These materials are provided 'AS IS' WITHOUT WARRANTY OF ANY KIND, EITHER EXPRESSED OR IMPLIED, INCLUDING, BUT NOT LIMITED TO, THE IMPLIED WARRANTIES OF MERCHANTABILITY, FITNESS FOR A PARTICULAR PURPOSE, OR NON-INFRINGEMENT. TransCelerate and its members do not accept any responsibility for any loss of any kind including loss of revenue, business, anticipated savings or profits, loss of goodwill or data, or for any indirect consequential loss whatsoever to any person using these materials or acting or refraining from action as a result of the information contained in these materials. Any party using these materials bears sole and complete responsibility for ensuring that the materials, whether modified or not, are suitable for the particular use and are accurate, current, commercially reasonable under the circumstances, and comply with all applicable laws and regulations.

Nothing in this template should be construed to represent or warrant that persons using this template have complied with all applicable laws and regulations. All individuals and organizations using this template bear responsibility for complying with the applicable laws and regulations for the relevant jurisdiction.

The Core Backbone of the CSR Template

The **Core Backbone** contains CSR information common to all phases, study populations, and therapeutic areas. The core backbone is streamlined and focused on reporting the results of the study without detailed interpretation of the general benefit and risk of the study intervention, which should be reserved for CTD module summary documents.

Core Backbone Headings

- Do not rearrange or reorder sections of the CSR template.
- Level 1 headings should be consistent across CSRs that use the TransCelerate Common CSR Template for reference and mapping purposes.
- Level 1 headings should not be deleted. If they are not relevant to the study, "Not applicable" should be inserted so that the numbering of subsequent sections is not changed.

Commented [BWG1]: In addition to the comment boxes, some track changes are included throughout the template to improve readability.

Commented [BWG2]: Suggest to provide the contact information (or at a minimum an email address) where comments can be submitted.

Commented [BWG3]: The term 'Core' is not related to 'CORE Reference' and should not be confused with it.

Commented [BWG4]: It is not clear if this means the template is applicable to non-interventional studies. Note: CORE Reference is specifically limited to interventional studies.

Commented [BWG5]: Manuscript Table 1 Major Critical Review Finding:
A single CSR template cannot fit all study designs without inherent structural flexibility. Thus there should be the ability to rearrange sections to suit the study design. We think that that the template's Level 1 headings would fit any study design but there should be the ability to rearrange Level 2 headings and below. Also the lack of structure with regard to what used to be Section 14 and Appendix 16 was not appreciated by the BWG.

The CSR author must also consider the practical value to enhance the reviewability of the document by the Regulatory Reviewer. If the Regulatory Reviewer is accustomed to finding information in a particular location (at least with respect to Level 2 headings), as identified by heading numbering sequence, then inflexibility is beneficial; however, if the ordering/deletion of sections becomes an impediment that merely confounds reporting and review, then more flexibility should be allowed.

© 2016, 2018 TransCelerate BioPharma

- Level 2 and lower headings can be deleted/added/modified as needed, however do not rearrange or reorder sections.

Terminology

- The following terminology has been selected for use within TransCelerate common templates (protocol, statistical analysis plan [SAP], and CSR) and is considered to be appropriate for all phases, study populations, and therapeutic areas.
  - *Participant* is used rather than subject, healthy volunteer, or patient.
  - *Study intervention* is used rather than study drug. Study intervention covers all types of investigational and non-investigational products including medical devices and vaccines.
    - Study intervention is defined as investigational intervention(s), marketed product(s), placebo, or medical device(s) intended to be administered to a study participant per protocol.
- *Effectiveness* is used for Medical device studies whereas for drugs, *efficacy* is used. Template should be updated as appropriate.

Commented [BWG6]: The BWG agree on this definition.

Commented [BWG7]: The BWG agree on this definition however, we suggest it should be acceptable to modify it depending on the study design. Phrases such as “withdrawal of intervention” can also be considered awkward. Additionally, in some studies there may be an investigational drug and an investigational device (for example) so different terms may be useful.

Commented [BWG8]: It is unclear if this then precludes observational studies or those involving historical control comparisons.

Formatting and Text Conventions

- Common Headings: Heading levels 1 should not be altered, deleted (indicate “not applicable” if needed), or rearranged.
- Suggested Headings: Heading levels 2 and lower are suggested and may be modified or deleted as necessary, however do not rearrange or reorder sections.
- Common Text: Black font preceded by <Start of common text> and followed by <End of common Text> is common language intended to be harmonized across protocols. The recommendation is to use this text as written to maintain consistency across template users, but the text can be adapted if required. The flags for the start and end of common text can be removed automatically at the time of CSR finalization if the technology enabled CSR template has been used, or should be removed manually by the author.
- Suggested Text: Black text preceded by <Start of suggested text> and followed by <End of suggested Text>that is not flagged as common text is suggested language to be used in optional sections and can be deleted/modified as needed. The flags for the start and end of common text can be removed automatically at the time of CSR finalization if the technology enabled CSR template has been used, or should be removed manually by the author.
- Variable Text: Blue bracketed text is variable text that should be ~~addressed~~ applied based on individual study needs.
- *<Start of suggested text>* Green italicized text is *<Start of suggested text>* and should be removed by the author if not utilized.
- Instructional Text: Red text is intended to aid in authoring of the CSR in this template. In the Basic Word Edition, it is red, hidden text, and paragraph marks must be enabled in order for it to be displayed. In the Technology-Enabled Edition, it will appear only in the Instructional Text panel.

Commented [BWG9]: This seems to duplicate the instructions on the previous page.

Commented [BWG10]: As per earlier comments, we suggest that in some cases level 2 headings and lower may need to be rearranged or reordered.

Disclosure

- Users are reminded that many regulators have begun to require increased public disclosure of clinical trial documents. For recommendations related to using this template given the disclosure requirements, please refer to the best practices for disclosure in the implementation toolkit available on TransCelerate's website.

Appendices and Tables/Listings/Figures

- Reference ICH E3 and CORE (**C**larity and **O**penness in **R**eporting: **E**3-based) for guidance on the naming/numbering of tables/listing/figures and appendices. As the Common CSR template headings end at Section 7 (References), number of tables/listing/figures and appendices could be numbered Section 8 and 9, respectively.
- Consider providing the principal/coordinating investigator name and contact information in the List and Description of Investigators and Other Important Participants appendix for privacy purposes.
- In alignment with CORE Reference, recommendation is to include signatory pages as an appendix.

Commented [BWG11]: It would be helpful to include a link to this resource.

Commented [BWG12]: Manuscript Table 1 Major Critical Review Finding:
In lieu of providing CSR appendices templates, this template points to ICH E3 and CORE Reference for guidance. However, ICH E3 and CORE Reference direct links are not included in the template. In future iterations of the TransCelerate CSR template, we would like to see the user experience enhanced by addition of direct links in the template instructional text:
- www.core-reference.org (website where CORE Reference may be downloaded from)
- https://www.ich.org/products/guidelines/efficacy/article/efficacyguidelines.html (the ICH efficacy guidelines webpage where ICH E3 may be downloaded from).

Commented [BWG13]: The lack of numbering of the first several CSR sections skews later numbering and conflicts with ICH E3, other guidances, and CORE Reference (which refers to E3 Sectional numbering in which Appendices start at "16" with "16.1.1. Protocol and protocol amendments"). However, the instructions state "refer to E3 and CORE Ref for numbering convention" but it is not possible to follow E3/CORE Reference numbering when following this template numbering.

Commented [BWG14]: For EMA and FDA jurisdictions, the Principal Investigator and Coordinating Investigator names are already in the public domain in the clinical trial registry information (EUDRACT and clinicaltrials.gov) so the Principal Investigator and Coordinating Investigators names can be included but the contact details would need to be redacted if included or provided in an appendix instead. Details of all other 'important participants' should be placed in an appendix to reduce the need for later redaction.

Commented [BWG15]: The FDA guidances do not refer to the term "Principal Investigator", although other US government agencies do (e.g., NCI). "Coordinating Investigator" is a term in ICH E6.

Commented [BWG16]: We suggest to mention these points here:
- In the EU, Appendices 16.1.1, 16.1.2, and 16.1.9 will be publicly disclosed, so anything included in these appendices should conform to current minimum standards for de-identifying data
- In the US pilot program, Appendices 16.1.1 and 16.1.9 will be publicly disclosed.

Commented [BWG17]: Manuscript Table 1 Major Critical Review Finding:
Instructional text is missing around transparency & disclosure; however, TransCelerate do reference their 'implementation toolkit' under the Disclosure heading on the previous page so authors should use that along with the template. However, as noted above, a link to the toolkit here would be useful because the TransCelerate website is not easy to navigate and could deter users who may not have time to search for particular referenced documents (link: https://www.transceleratebiopharmainc.com/assets/clinical-data-transparency/).

© 2016, 2018 TransCelerate BioPharma

Title Page

**Study Title:**	
**Short Title:**	
**Study Intervention:**	Provide Compound name/number, Established (generic) name and/or Device name as provided in the protocol.
**Trade Name:**	If available, trade name can also be provided, or row may be deleted
**Indication:**	Delete this row if not applicable.
**Brief Description:**	Provide a brief (one to two sentences) description giving design (parallel, crossover, blinded~~ing~~, randomized), comparison (placebo, active, dose/response), duration, dose, and participant population. Delete this row if not applicable (e.g., details provided in Study Title).
**Study Sponsor:**	Per CORE Reference, suggest excluding 'CSR contact/signatory' from title page to protect privacy. To ensure that the 'CSR contact' remains linked to the CSR, suggest including details in an appendix<br>.
**Study Number:**	
**Study Phase:**	
**Study Initiation Date:**	[DD Month YYYY] ([first signed informed consent/first participant first visit/enrollment/randomization])
**Early Study Termination Date:**	Delete this row if not applicable.<br>[DD Month YYYY] ([last participant last visit/date of last observation from last participant])<br>The analyses presented in this report are based on a database lock date of [DD Month YYYY].

Commented [BWG18]: Is this meant to apply only to the product under investigation, or does it include any product (e.g., comparator, placebo) used to assess comparative efficacy/safety? Also how does this apply for non-interventional studies?

Commented [BWG19]: Are there any cases when 'indication' would not be applicable? Even exploratory studies are intended to pursue information on effectiveness/safety in a condition-of-interest.

Commented [BWG20]: Although the Sponsor Contact/Signatory is usually provided, it is unclear how this differs from the CSR Contact/Signatory. Also suggest that the phone or fax numbers are not included since they would need to be redacted later on.

Commented [BWG21]: This should be placed next to the study title.

Commented [BWG22]: We agree this should be flexible, but the milestone should be indicated (e.g., indicate first signed informed consent form, if that is what is used).

© 2016, 2018 TransCelerate BioPharma

**Primary Completion Date:**	Provide primary completion date as defined in the protocol. Delete row if not applicable.<br><br>[DD Month YYYY] ([last participant last visit/date of last observation from last participant])
**Study Completion [OR] Interim Data Report Date:**	Delete row if not applicable. Select appropriate header (either study completion or interim data report).<br><br>[DD Month YYYY] ([last participant last visit/date of last observation from last participant/date of interim data cut-off])<br>The analyses presented in this report are based on a database lock date of [DD Month YYYY].
**Regulatory Agency/Registry Identifier Number(s):**	Regulatory Agency Identifier Number(s) (e.g., EuDRA CT, NCT) are to be provided here. Pediatric investigational plan and/or pediatric study plan number(s) may also be added here if applicable.
**Report Date:**	Enter CSR version (e.g., original, primary, interim, final, supplemental) and indicate any earlier versions. Provide date that CSR was approved.<br><br>**Document Version** / **Date**<br>Enter CSR Version /

Commented [BWG23]: This term might be confusing. Suggest an explanatory note is inserted into the instructions whenever clarification would be valuable. For example: "The Primary and Study Completion Dates may be the same date, but they have different definitions. The Primary Completion Date is based on the data collection for the Primary Outcome Measure. The Study Completion Date is based on the last data collection point for the last participant".

Commented [BWG24]: For an interim analysis, date of data cut-off (as used in CORE Reference) is more important than database lock date.

Commented [BWG25]: Manuscript Table 1 Major Critical Review Finding: An 'ICH GCP statement" such as "This study {was/was not} conducted in compliance with International Council for Harmonisation (ICH) Good Clinical Practice (GCP), including the archiving of essential documents" is missing. Regulatory auditors are known to question how the claim of study conduct in compliance with GCP is supported, so it is important such a statement is included. We also suggest that a reference to compliance with the Declaration of Helsinki (with specific reference to version/date) is added.

Synopsis

A brief stand-alone synopsis without cross-reference to other sections of the CSR or other documents (suggested length of 6 to 7 pages) that summarizes the study should be provided. In addition to a brief description of the study design and critical methodological information (what was actually done), the synopsis should provide a summary of all relevant results (e.g., if there are multiple endpoints, consider limiting to primary and secondary) obtained during the study, as well as other critical information, including data on the study population, disposition of participants, and study intervention compliance. The synopsis should include numerical data to illustrate results, not just text or p-values (consider presenting results as summary tables to reduce the amount of text in the synopsis). Do not include personally identifiable information such as participant IDs, demographic data (race, sex, and age) and medical history (e.g., rare diseases) of individual participants.

Name of Sponsor/Company:

Name of Study Intervention:

Provide Compound name, Established (generic) name, and/or Device name as provided in the protocol.

Trade Name:

If available, trade name may also be provided. Delete this section if not applicable.

Study Title:

Study Number:

Study Phase:

Provide study phase as shown on title page.

PIP and/or PSP number (if applicable):

Provide pediatric investigational plan (PIP) and/or pediatric study plan number(s) if applicable. Delete heading if not applicable.

Number of Study Center(s) and Countries:

Insert the number of centers, participants (e.g., enrolled/randomized/screened), and add the respective countries and/or regions. If list of countries is extensive, consider providing the number of countries rather than listing them individually. Do not include center IDs, individual center Investigator names or addresses here; protected personal data may be provided in an appendix (e.g., List of Investigators).

This study was conducted at [X] centers that [enrolled/randomized/screened] participants in [specify countries or regions].

Commented [BWG26]: CORE Reference suggests 3 pages, although longer is fine for more complex studies, so a limit of 6 to 7 pages seems reasonable.

Commented [BWG27]: Inclusion of 'important protocol deviations' (as per E3 2012 Q&A) is missing here.

Commented [BWG28]: E3 2012 Q&A states that "use of a tabular format synopsis is not mandatory" which would be useful to include here as instructional text to allow flexibility.

Commented [BWG29]: For transparency and disclosure reasons, we suggest including the inserted instructional text opposite (or similar text).

Commented [BWG30]: If it is appropriate to provide the PIP number, why not include the IND number?

Commented [BWG31]: We agree that Individual Principal Investigator/Coordinating Investigator name is not necessary here since it is already given on the title page. However, it is of note that ICH E3 guidelines require the name of the Principal or Coordinating Investigator in the CSR Synopsis.

Commented [BWG32]: The template text needs review and editing since capitalization is inconsistent e.g., investigator/Investigator. Use of periods, spacing etc. are also inconsistent In the template.

© 2016, 2018 TransCelerate BioPharma

Publications (if any):

List any publications – including abstracts or posters – of the study, as well as publications describing interim or post-hoc analyses. Only include publications that are available i.e., published not just accepted.

Study Period:

Provide the study initiation date, and the applicable end date (e.g., early termination/primary completion/study completion/interim report date) as shown on title page.

Methodology:

Provide details around overall study design from the protocol (e.g., protocol synopsis). Include additional detail or edit, as appropriate to briefly summarize the study design, including design, study intervention groups/cohorts, and critical methodological information.

Number of Participants (planned and analyzed):

Describe the number of planned participants as stated in the protocol and the actual number of participants [e.g., randomized, treated]. Provide brief summary of the number of participants analyzed per analysis population (e.g., evaluable, PK, safety).

Diagnosis and Main Criteria for Inclusion and Exclusion:

Provide a high-level summary only of the key eligibility criteria that define the participant population – do not list all inclusion/exclusion criteria.

Study Interventions, Dose, Mode of Administration, and Batch Number(s):

Briefly describe the study intervention(s) (e.g., comparator, placebo, medical device). Do not detail the components of the formulation (unless critical to explain study design). Batch numbers may be provided in this section or for transparency, ~~i~~they may be provided in the Drug Manufacturing Lot Number appendix.

Duration of Study Intervention:

Commented [BWG33]: In CORE Reference, we use 'Reporting period' since this is considered to be clearer e.g., for an interim analysis, 'Study Period' is not strictly correct.

Commented [BWG34]: Some terminology guidance might be helpful and perhaps an associated glossary could be referenced (e.g., CDISC Annex or URL).

Commented [BWG35]: Manuscript Table 1 Major Critical Review Finding: 'Background and rationale for the study' is missing from the Synopsis. We suggest that this is included (per CORE Reference) since this is required for EU posting per CTR EU 536-2014.

Commented [BWG36]: This is a useful addition.

Commented [BWG37]: In CORE Reference this is split into test product and control product which makes it easier to see the main product of interest rather than having them all together here.

Commented [BWG38]: If there is a post-intervention period, it is not clear where this should be addressed.

© 2016, 2018 TransCelerate BioPharma

Objectives, [Estimands/]Endpoints, Statistical Methods and Results

From the protocol, provide the Objectives and Estimands/Endpoints table (in alignment with ICH E9 (R1) guidance) or the Objectives and Endpoints table (if estimands were not defined for the study). Recommendation is to provide only primary and secondary objectives/estimands (and/or endpoints) in the synopsis. Other (exploratory/tertiary) endpoints may be provided in the body; critical exploratory/tertiary endpoints may be elevated to the synopsis if applicable.

Two sample tables are provided in the template; please select the appropriate table based on whether the protocol provided estimands/endpoints or only endpoints:

Example Table 1: Insert the Objectives and Estimands/Endpoints table from the protocol into this section. Insert 2 columns to the right of the Estimands/Endpoints column (see Example Table 1) and provide a high-level description of the statistical analyses methods and the results for the primary and secondary estimands/endpoints. Note that studies may have both estimands and endpoints: estimands for objectives that are formally analyzed, and endpoints for objectives for which the data are summarized descriptively (e.g., safety objectives such as AEs, routine laboratory assessments) (see Example Table 1). A Statistical Methods section may be added to the Synopsis if sufficient critical information cannot be provided in this table.

Example Table 2: If endpoints (ie, not estimands) were provided in the protocol, insert the Objectives and Endpoints table from the protocol into this section. Insert 2 columns to the right of the Objectives and Endpoints table (see Example Table 2) and provide a high-level description of the statistical analysis methods and results for the primary and secondary endpoints. A Statistical Methods section may be added to the Synopsis if critical information cannot be provided in this table.

Listed below are the objectives and [estimands and/or endpoints] that are described in this report.

Example Table 1:

Objectives	Estimands/Endpoints	Statistical Analyses	Results
Primary			
To compare the efficacy of [study intervention] with [active control] in	Primary Estimand:	ANCOVA with reference-based multiple imputation	Noninferiority of [study intervention] against [active control] was demonstrated with respect

Commented [BWG39]: Manuscript Table 1 Major Critical Review Finding: Objectives of the study should be apparent before reading the study methodology etc. so that these can be placed into context. In this document, we suggest that they occur too far down the order to support comprehension so we suggest that this table is placed earlier in the Synopsis.

Commented [BWG40]: We are concerned that this table could become long and cumbersome for complex studies, especially if all exploratory endpoints are to be included.

Commented [BWG41]: CORE Reference suggests to include ALL endpoints in the Synopsis to support comprehension and transparency. However, it was considered possible that there could be some occasions where it might not be relevant to include all exploratory endpoints in the Synopsis. In such cases, we suggest that an instruction that only critical endpoints should be included could lead to selective presentation of exploratory results. The statistician commented that exploratory endpoints are likely to be underpowered and any conclusions drawn are likely to be speculative so we suggest that a statement such as "Exploratory endpoints are likely to be underpowered and any conclusions drawn are likely to be speculative" would be useful to include.

Commented [BWG42]: We suggest that some guidance would be useful regarding the circumstances under which an estimand is unlikely to be defined in the protocol (e.g. early Phase 1 studies may not include estimands).

Commented [BWG43]: Manuscript Table 1 Major Critical Review Finding: The results column seems misplaced since this is the methodology part of the Synopsis so we suggest that the results column is deleted.

© 2016, 2018 TransCelerate BioPharma

participants with [indication] with respect to [objective of interest]	• Variable: Change from baseline in [clinical variable] at [timepoint]<br>• Population: FAS<br>• IES:<br>○ Initiation of rescue medication: "had rescue medication not been initiated" (hypothetical)<br>○ Discontinuation of study intervention due to adverse event: "regardless of study intervention discontinuation due to adverse event" (study intervention policy)<br>• PLS: Mean difference between interventions		to change from baseline in [clinical variable] at [timepoint] (estimated difference: x.x, 95%CI [x.x;x.x], p [0.XXX)
	Secondary Estimand:<br>• Variable:<br>• Population:<br>• IES:<br>• PLS:		
Secondary			

To compare the safety of [study intervention] with [active control] in participants with [indication]	Presence of TEAEs	Number and proportion of participants with at least one TEAE, number of events and number per 100 participant-years-of-exposure	Incidence of TEAEs was similar between [study intervention] and [active control]
•	•	•	•
CFB ANCOVA = change from baseline analysis of covariance; IES = Intercurrent event(s) strategy; PLS = Population-level summary; TEAE = treatment-emergent adverse event.<br>Exploratory endpoints are likely to be underpowered and any conclusions drawn are likely to be speculative.			

Commented [BWG44]: The suggested footnote could be added if exploratory endpoints are included in the table.

End of Example Table 1

Example Table 2:

Objectives	Endpoints	Statistical Analyses	Results
Primary			
To compare the efficacy of [study intervention] against [active control] in participants with [indication] with respect to [objective of interest]	Primary: Change from baseline in [clinical variable] at [timepoint]	ANCOVA with reference-based multiple imputation	Noninferiority of [study intervention] against [active control] was demonstrated with respect to change from baseline in [clinical variable] at [timepoint] (estimated difference: x.x, 95% CI: [x.x;x.x], p [0.XXX)
Secondary			
To compare the safety of [study intervention] and [active control] in participants with [indication]	Presence of TEAEs	Number and proportion of participants with at least one TEAE, number of events and	Incidence of TEAEs was similar between [study intervention] and [active control]

© 2016, 2018 TransCelerate BioPharma

		number per 100 participant-years-of-exposure	
Exploratory endpoints are likely to be underpowered and any conclusions drawn are likely to be speculative.			

End of Example Table 2

Commented [BWG45]: The suggested footnote could be added if exploratory endpoints are included in the table.

Summary of Results and Conclusions:

If results are reported in Objectives/Estimands (and/or Endpoints)/Statistical Analyses/Results table, suggest only sparse content be provided in this section (e.g. Demography and Baseline Characteristics, Exposure, and summary table of adverse events) and deletion of subsections already presented above to avoid duplication. Delete any subheadings that are not applicable to the study.

Recommendation is to provide only primary and secondary objectives/estimands (and/or endpoints) and key safety results in the synopsis. Other (exploratory) endpoints may be provided in the body; critical exploratory endpoints may be elevated to the synopsis if applicable. May include summary tables. If an estimand/endpoint was not analyzed, or results were not available at the time of the report, this should be stated. Post-hoc results and conclusions may be included, but must be clearly identified as being post-hoc with appropriate rationale.

Demography and Baseline Characteristics:

For studies with sites in the EU (for compliance with EU CTR), include the population of participants (including actual number of participants included in the clinical trial in the Member State concerned, in the Union, and in third [non-EU/EEA] countries); age group breakdown, gender breakdown, as applicable. Omit for non-EU studies or if no direct synopsis posting.

Exposure:

Efficacy Results:

Pharmacokinetic Results (if applicable):

Pharmacodynamic Results (if applicable):

Other Results (if applicable):

May include immunogenicity, biomarker, health economics, genetics results if applicable. Header may be edited to describe specific results, or subsections (or separate paragraphs) may be added if more than one type of result. Delete section if not applicable. If post-hoc results are included, they must be clearly identified as being post-hoc.

Commented [BWG46]: See earlier comment about removing the results column from the tables, in which case more detail on the results needs to be included here.

Commented [BWG47]: As above, it is suggested that all endpoints are included to avoid selective inclusion (or exclusion of poor or negative results).

Commented [BWG48]: This term should not be used - instead use the term "non-EU/EEA".

Commented [BWG49]: The sequence of results summaries should match the main body of the CSR.

Commented [BWG50]: Although the template suggests summary tables are used, we suggest that most readers prefer some text as well (i.e. to 'tell the story'). However, tables can be included to present much of the data.

© 2016, 2018 TransCelerate BioPharma

Safety Results:

Suggest reuse of summary table from Section 6.1.1 Brief Summary of Adverse Events and editing/deleting to remove non-critical information and provide simplified description of adverse events for the synopsis. An example table is provided.

<Start of suggested text>

	Study Intervention [1] (N = XX) n (%)	Study Intervention [2] (N = XX) n (%)
All treatment emergent adverse events	n (%)	n (%)
Treatment-related AEs	n (%)	n (%)
Fatal AEs	n (%)	n (%)
SAEs	n (%)	n (%)
Treatment-related SAEs		
AEs leading to discontinuation of IP	n (%)	n (%)
AEs leading to discontinuation from study	n (%)	n (%)
AEs of interest	n (%)	n (%)

<End of suggested text>

Conclusions:

Conclusions should be consistent with those in Section 7, focusing on estimands/endpoints described in the synopsis (e.g., primary and secondary).

Date and Version of This Report:

Provide date and version as stated on title page. Include any earlier reports from the same study by date, as applicable.

Commented [BWG51]: This section needs to allow for safety information other than AEs to be included, especially where safety is the primary objective of the study.

Commented [BWG52]: This link is incorrect and should be to Section 5.2.1.1.

Commented [BWG53]: The term "intervention" should be used, not 'IP'.

Commented [BWG54]: CORE Reference makes the distinction between discontinuing study intervention and the participant withdrawing from the study as a whole. We suggest that these differential terms aid comprehension rather than applying 'discontinue' for both.

Commented [BWG55]: This should be 'Section 6'.

© 2016, 2018 TransCelerate BioPharma

Table of Contents

The table of contents should include:

- The page number or other locating information of each CSR text section, including tables and figures embedded in the text (in-text tables and figures)
- During publishing of the CSR, the list and locations within the CSR of appendices, tabulations and any case report forms (CRFs) should be provided below the Table of Contents. Recommendation is to follow ICH E3 and CORE guidance for ordering/naming of these items as applicable to the study.

Title Page 5

Synopsis 7

Table of Contents 15

List of Abbreviations and Definitions of Terms 18

Ethics 19

1. Introduction 21

2. Study Objectives and [Estimands [and/or] Endpoints] 22

3. Investigational Plan 23
    3.1. Overview of Study Design 23
        3.1.1. Discussion of Study Design 23
        3.1.2. Change(s) in Study Conduct 24
    3.2. Investigators and Study Administrative Structure 25
    3.3. Selection of Study Population 25
        3.3.1. Inclusion/Exclusion Criteria 25
        3.3.2. Removal of Participants From Intervention or Study 25
    3.4. Study Intervention 25
        3.4.1. Study Interventions Administered 25
        3.4.2. Measures to Minimize Bias 27
        3.4.3. Study intervention Compliance 28
        3.4.4. Prior, Concomitant, [and/or] Post-intervention Therapy 28
    3.5. Study Assessments and Procedures 28
        3.5.1. Planned Measurements and Timing of Assessments 28
        3.5.2. Appropriateness of Measures 28
        3.5.3. Additional Summary of Specific Assessments 28

© 2016, 2018 TransCelerate BioPharma

3.6. Data Quality Assurance 28
3.6.1. Study Monitoring 29
3.6.2. Investigator Meetings and Staff Training 29
3.6.3. Standardization of Laboratory Procedures 29
3.6.4. Investigator Responsibilities 29
3.6.5. Clinical Data Management 30
3.6.6. Clinical Quality Assurance Audits 30
3.7. Statistical Analysis 30
3.7.1. Statistical Analysis Plan 30
3.7.2. Changes in Planned Analyses Prior to Unblinding or Database Lock 31
3.7.3. Changes Following Study Unblinding/Database Lock and Post-hoc Analyses 31
4. Study Participants 32
4.1. Disposition of Participants 32
4.2. Protocol Deviations 34
4.3. Populations Analyzed 34
4.4. Demographic and Other Baseline Characteristics 35
4.5. Prior, Concomitant, [and/or] Post-intervention Therapy 35
4.6. Exposure and Study Intervention Compliance 35
4.6.1. Exposure 35
4.6.2. Dose Modification 35
4.6.3. Measurement of Compliance 35
5. Evaluation of Response to Study Intervention 36
5.1. Efficacy 36
5.2. Safety 37
5.2.1. Adverse Events 37
5.2.2. Clinical Laboratory Evaluation 39
5.2.3. Other Safety Evaluations 40
5.3. Pharmacokinetics 41
5.4. Pharmacodynamics 41
5.5. Genetics 41
5.6. Biomarkers 41
5.7. Immunogenicity 41
5.8. [Health Economics] OR [Medical Resource Utilization and Health Economics] 41
5.9. [Other] 42
5.10. Summary of Evaluation of Response to Study Intervention 42

© 2016, 2018 TransCelerate BioPharma

6. Conclusions 43

7. References 44

Commented [BWG56]: The 'Ethics Section' is not listed here. Also, other than exclusion of some section numbers, the absence of an 'Administrative Structure' section and inclusion of all the results under a single major section, there are not too many differences in the high level organization compared to ICH E3.

As such, we suggest that it is not worth revising the Level 1 headings and they should remain as per ICH E3 and CORE Reference.

Commented [BWG57]: No 'Table of Contents' for in-text tables and figures is provided but these are useful to have in the template to remind authors to include them.

List of Abbreviations and Definitions of Terms

A list of the abbreviations and definitions of specialized or unusual terms or unusual measurements units should be provided. Abbreviations do not need to be specified on first mention in the text. Common abbreviations (e.g. UK, USA) need not be defined. In the case where an abbreviation is the same for two different terms, e.g. American Diabetes Association (ADA) and Antidiabetic Agents (ADAs), one of the two terms should be written out in full in all instances to avoid any confusion.

Example:

ABBREVIATIONS

AE Adverse event

DEFINITIONS OF TERMS

QT interval The portion of an electrocardiogram between the onset of the Q wave and the end of the T wave.

Abbreviation or Term	Definition/Explanation
[abbreviation or term]	[definition/explanation]

Commented [BWG58]: For the CSR template, TransCelerate places the list of abbreviations at the beginning, but for the TransCelerate protocol template, abbreviations are appended. The BWG suggest it is better to have them placed here at the beginning.

Commented [BWG59]: This is a departure from usual practice and could hinder readability since it can be time-consuming and irritating to have to constantly refer back to the abbreviations list to find out what a term means, whereas if it is spelt out in full on first use, the reader knows what it means without interrupting the reading flow.

Commented [BWG60]: We suggest that the glossary might sit better in an appendix.

Ethics

Independent Ethics Committee and/or Institutional Review Board

It should be confirmed that the protocol and any of its amendments, as well as information provided to participants and any recruitment advertisements, etc. were reviewed by an Independent Ethics Committee (IEC) and/or Institutional Review Board (IRB). A list of all IECs/IRBs consulted should be given in Appendix [X.X.X] List of IECs and IRBs and Representative Written Information for Participant and Sample Consent Forms and, if required by the regulatory authority, the name of the committee Chair should be provided.

<Start of suggested text>

- The protocol, protocol amendments, ICF, Investigator Brochure, and other relevant documents (e.g., advertisements) were submitted to an IRB/IEC by the investigator and reviewed and approved by the IRB/IEC before the study was initiated.
- Any amendments to the protocol required IRB/IEC approval before implementation of changes made to the study design, except for changes necessary to eliminate an immediate hazard to study participants.

<End of suggested text>

Ethical Conduct of the Study

It should be confirmed that the study was conducted in accordance with the ethical principles that have their origins in the Declaration of Helsinki and in accordance with ICH GCP.

<Start of suggested text>

This study was conducted in accordance with the protocol and with the following:

- Consensus ethical principles derived from international guidelines including the Declaration of Helsinki and Council for International Organizations of Medical Sciences (CIOMS) International Ethical Guidelines
- Applicable ICH Good Clinical Practice (GCP) Guidelines
- Applicable laws and regulations

<End of suggested text>

Participant Information and Consent

Briefly describe how and when informed consent was obtained in relation to participant enrolment (e.g. at pre-screening, enrollment).

<Start of suggested text>

Commented [BWG61]: It seems strange that this section is not numbered. All others are, so the lack of numbering here stands out.

Commented [BWG62]: As the use of ePRO interfaces becomes more common, there is also a need to provide to the IRB/IEC the screens that will be accessed by the participants to ensure that they are intuitive. We are not sure if this qualifies under "information provided".

Commented [BWG63]: ICH E3 has this as Appendix 16.1.3. It is of note that the TransCelerate template does not provide any guidance to the appendix organisation so this would be a useful addition.

Commented [BWG64]: There is no clarification that these do not need to be included since they are in the TMF and can be provided on request (in line with ICH E3 2012 Q & A Point 3).

Commented [BWG65]: This would be easier to read as paragraphs rather than bullet points.

Commented [BWG66]: Although this circumvents having to include the version/year of the Declaration of Helsinki, it was felt that the version and date should be included, since often, the most current iteration is not the version that was followed, or it may be that an earlier version was in effect at the initiation of a long-term study. There are also situations (e.g., Declaration of Helsinki clauses about use of placebo) that some regulators do not agree to so a different version may have to be used.

Commented [BWG67]: These are international ethical guidelines for health-related research involving humans: https://cioms.ch/wp-content/uploads/2017/01/WEB-CIOMS-EthicalGuidelines.pdf but most of the BWG have never seen these referenced in a CSR.

Commented [BWG68]: There should also be a section that describes special measures taken to protect vulnerable participants, when applicable. This is articulated in the Declaration of Helsinki and in regulatory guidance.

- The investigator or his/her representative explained the nature of the study to the participant or his/her legally authorized representative and answered all questions regarding the study.

- Participants were informed that their participation was voluntary. Participants or their legally authorized representative were required to sign a statement of informed consent that met the requirements of 21 CFR 50, local regulations, ICH guidelines, Health Insurance Portability and Accountability Act (HIPAA) requirements, where applicable, and the IRB/IEC or study center.

- The medical record included a statement that written informed consent was obtained before the participant was enrolled in the study and the date the written consent was obtained. The authorized person obtaining the informed consent also signed the ICF.

- Participants were re-consented to the most current version of the ICF(s) during their participation in the study.

- A copy of the ICF(s) was provided to the participant or the participant's legally authorized representative.

<End of suggested text>

Commented [BWG69]: This section would be easier to read as paragraphs rather than bullet points. Bullet points should only be used where there is a list of items.

Commented [BWG70]: Manuscript Table 1 Major Critical Review Finding:
As US laws/acts are cited, we suggest that equivalents in other jurisdictions should be included for consistency. However, this might be cumbersome in multi-country studies so the wording here should be more general e.g. '…local laws and applicable data privacy regulations'.

Commented [BWG71]: Manuscript Table 1 Major Critical Review Finding:
Reference should be made to the location of the master Patient Information Sheet and the Informed Consent Form being in the TMF.

1. Introduction

Provide the Study Rationale information from the protocol in this section; if content is excessively long, trim text to provide only the relevant sections (target length 1-3 pages). Reference any external guidelines that were followed and/or a high-level summary if specific health authorities were consulted in designing the study (avoiding commercially confidential information [CCI] such as meeting minutes or advice from a health authority that would be better reserved for CTD documents). Provide additional new information since the start of the study (e.g., safety updates, early termination) and an explanation of partial reporting (e.g., biomarkers to be provided later as addenda). Especially for studies with multiple consecutive analyses and reports, the introduction should contain a clear statement on which cut-off date(s) is(are) used for the analyses reported (e.g. date of database release).

Commented [BWG72]: This instruction implies that 'Study rationale information' is all that is needed – and it may therefore be inferred that background information on the disease and current treatments - and hence the medical need for the new study intervention – are all not needed.

CORE Reference provides a more rounded Introduction that tells the 'story' of the study.

Commented [BWG73]: The BWG team often encounter the controversial debate as to whether the CSR introduction should review the new literature published after protocol finalization. As the study was performed without the new literature, we suggest new literature should not be included. It would be helpful if guidance could be provided on this point.

© 2016, 2018 TransCelerate BioPharma

2. Study Objectives and [Estimands [and/or] Endpoints]

From the protocol, provide the Objectives and Estimands/Endpoints table (in alignment with ICH E9 (R1) guidance) or the Objectives and Endpoints table (if estimands were not defined for the study). Whereas only the primary and secondary objectives and estimands (and/or endpoints) are presented in the Synopsis, this section may also provide relevant tertiary/exploratory objectives and endpoints. If exploratory objectives reveal plans and/or possible future indications for the product, they should be provided in a separate subsection to ease redaction/anonymisation.

The objectives and estimands (and/or endpoints) should be per protocol (and any global amendments).

If an objective is not addressed in the CSR, it may be excluded from this section (in this case, manually delete the objective and its related estimand (and/or endpoint). However, its absence should be explained in Section 3.1.2 Changes ~~to~~ in Study Conduct or Section 3.7.2 Changes in Planned Analyses.

<Start of suggested text>

Objectives	[Estimands/Endpoints]
Primary	
•	•
Secondary	
•	•
Tertiary/Exploratory	
•	•

<End of suggested text>

Commented [BWG74]: Suggest that 'must' is used and not 'should' since all objectives and endpoints must be provided here.

Commented [BWG75]: Suggest adding this sentence to guide the author regarding disclosure considerations.

Commented [BWG76]: We suggest that even in such a case, the objective and endpoint should be stated here and then elsewhere in the CSR, it can be explained why the data are not provided, otherwise these appear as missing with no explanation until the end of the methods section.

Commented [BWG77]: The problem with the current table layout is that it does not allow for a secondary endpoint to be linked to a primary objective. Instructional text should be provided to help in such a circumstance.

© 2016, 2018 TransCelerate BioPharma

3. Investigational Plan

3.1. Overview of Study Design

Provide the study schema (if applicable) and description of overall design from the protocol in this section.

The study design is depicted below in Figure 1. Additional details are available in the protocol [Appendix [X.X.X] Study Protocol].

3.1.1. Discussion of Study Design

The scientific rationale from the protocol may be used in this section, including items such as those described below. Alternatively, for a more streamlined approach, provide only the sentence provided in this section that references the scientific rationale section of the protocol.

Summarize (briefly) study design based on main methodological features of study, such as the following if not provided in the section above:

- Single or multi-center, single or multi-country (avoiding inclusion of center IDs)
- Study interventions ~~studied~~ evaluated (drugs, doses, devices, and procedures)
- Population of participants studied and the number of participants to be included
- Level and method of blinding/masking (e.g., open, double-blind, single-blind, blinded evaluators and unblinded participants and/or investigators)
- Controls employed (e.g., placebo, no study intervention, active drug, dose-response, historical) and study configuration (parallel, cross-over)
- Method of assignment to study intervention (randomization, stratification)
- Sequence and duration of all study periods, including pre-randomization and post-treatment periods, therapy withdrawal periods and single- and double-blind treatment periods. When participants are randomized should be specified.
- Any safety, data monitoring or special steering or evaluation committees (if any relevant information is needed here it should remain as concise as possible avoiding the inclusion of any personally identifiable information)
- Any interim analyses
- Special features (e.g., handling of dropouts kept in the study, enrolling different types of participants at different centers, PK variables in selected participants, data from electronic health records).

References to the relevant figure and the protocol appendix are appropriate.

Commented [BWG78]: Manuscript Table 1 Major Critical Review Finding: The fundamental points needed to understand the study should be readily available in the CSR. Cross-references should be for additional information only and should not be used to link to important information in the protocol, otherwise the comprehension of the CSR as a stand-alone document is negatively impacted.

Commented [BWG79]: Per the previous comment, it is not sufficient to cross-reference to the protocol since the CSR should be a stand-alone document.

Commented [BWG80]: It may be applicable to note in this section if the study design is well established for the class of drug or indication.

Commented [BWG81]: Include this as instructional text.

Commented [BWG82]: In the EU, devices are now regulated under MEDDEV 2.7.1 Rev.4 which requires a clinical investigation report (CIR) – most commonly for devices, although a CSR can also be also acceptable. The CIR is the device equivalent report to a CSR for a drug/biologic. So for the EU, the mention here of 'device' is - we assume - for a drug/biologic-device combination – which must be reported in a CSR (not a CIR).

Commented [BWG83]: Include this as instructional text.

Commented [BWG84]: It may be helpful to emphasize that these could be planned or "for-cause" (e.g., to assess for futility).

Commented [BWG85]: Justification for use of any special features or unusual study design elements should be included.

© 2016, 2018 TransCelerate BioPharma

The scientific rationale for features of the study design, including chosen control group(s), dose(s), and endpoint(s), as applicable, are discussed in the Scientific Rationale for Study Design section of the protocol [Appendix [X.X.X] Study Protocol].

3.1.2. Change(s) in Study Conduct

Changes in the conduct of the study should be addressed in this section; changes in planned analyses are to be described in Section 3.7.2 Changes in Planned Analyses. Provide only a brief summary of substantial changes as detailed information is provided in the protocol amendments appended to the CSR. Changes in the conduct of the study can include, but are not limited to, dropping a treatment group, changes in entry criteria or drug (study intervention or non-study intervention) dosages, adjusting the sample size, and changes in assessment schedules. These changes would require a protocol amendment and can therefore be cross-referenced to the summary of protocol amendment changes, often included in Appendix [X.X.X] Study Protocol to avoid duplication. If a summary of the change is to be included in the CSR text, an overview of the changes and rationale for the changes should be included and may be presented in a tabular format. In the event any changes were made to the study conduct without a corresponding protocol amendment, then this summary should also include why it was agreed not to amend the protocol and the role that approved this decision.

In general, changes made prior to breaking the blind have limited implications for study interpretation. It is therefore particularly critical that the timing of changes relative to blind breaking and availability of outcome results are also well characterized. Any possible implications of the change(s) for the interpretation of the study should be discussed briefly in this section and more fully in other appropriate sections of the report. Do not include personnel changes to minimize unnecessary references to personal protected information (PPI).

It is expected that changes in study conduct would be made while the study is still blinded but this should be corroborated and confirmed in the CSR text. Blind-breaking may occur for regulatory purposes or medical purposes; check with the safety officer and statistician as to whether the blind was broken. If blind was broken, changes made after study unblinding should be described separately in Section 3.7.3 Changes Following Study Unblinding and Post hoc Analyses, and include the study intervention group, and the event leading to code breaking. Specify whether the Investigator or the Pharmacovigilance representative (for the purpose of expedited reporting) broke the blind.

Select appropriate options provided in the template.

[Option 1]: Changes in the conduct of the study that were implemented by protocol amendment(s) are described in Appendix [X.X.X] Study Protocol. Additional changes in the conduct of the study are described below.

[Option 2]: All changes in the conduct of the study were implemented by protocol amendment(s), as described in Appendix [X.X.X] Study Protocol.

[Option 3]: There were no changes in the conduct of the study.

Commented [BWG86]: Manuscript Table 1 Major Critical Review Finding: The CSR author cannot assume at reporting that all such information is properly documented/recorded in precursor documents such as the protocol. The CSR author needs to check carefully that all relevant information is provided and that there are no inadvertent omissions in the CSR because the section is missing in the protocol.

Commented [BWG87]: CORE Reference has this as Section 9.8.1 through 9.8.3 but the TransCelerate template has split these into 'Changes in study conduct' (placed here) and 'Changes in the planned analyses' which is within the statistics section. Since changes in the planned analyses may not always result from changes in study conduct - they often arise from emerging data (observed or literature) - it makes sense to collate analyses changes together with the planned statistical analysis section.

Commented [BWG88]: We agree that only substantial changes need to be noted - administrative changes do not.

Commented [BWG89]: We again encounter the issue of toggling between the CSR and the protocol - so it is important that changes are triaged according to the criticality of the change, so as not to de-emphasize something that is really critical to the understanding of the disease, intervention, analysis etc.

Commented [BWG90]: We suggest that only changes to the planned analyses after study unblinding should be described in Section 3.7.3 and changes in study conduct after study unblinding should be placed here.

Commented [BWG91]: Manuscript Table 1 Major Critical Review Finding: If there are 10 amendments, there could be 10 protocols in the appendix (or more commonly only the most recent one, with others available on request) so it would be tedious for a reviewer to have to search through each to determine what has changed.

© 2016, 2018 TransCelerate BioPharma

3.2. Investigators and Study Administrative Structure

Required information is to be provided in referenced appendices (e.g., include Sponsor and CRO contact information in the appendix that presents the list of investigators and other important study personnel). For a streamlined approach, suggest only providing text that directs the reviewer to the appropriate appendices. Do not include any personal protected information (PPI) or epatented measurement) in this section.

3.3. Selection of Study Population

3.3.1. Inclusion/Exclusion Criteria

Provide a high-level description of the study population (such as the description of the eligibility criteria/study population from the protocol synopsis) with a cross-reference to the protocol for the specific inclusion/exclusion criteria.

Commented [BWG92]: **Manuscript Table 1 Major Critical Review Finding:** The CSR should be somewhat independent, so that at least the key selection criteria should be itemized in the CSR. When reviewing the results, the reader may well wish/need to look back at the full in/exclusion criteria, but with this template, they would need to go back to the protocol, which is time-consuming.

Enrolled in this study were participants with [provide indication or population]. Detailed inclusion and exclusion criteria are provided in Appendix [X.X.X] Study Protocol.

3.3.2. Removal of Participants From Intervention or Study

Text referencing the appropriate section of the protocol is provided.

The specific criteria and procedures for early discontinuation from study intervention(s) or withdrawal from the study are described in Appendix [X.X.X] Study Protocol.

3.4. Study Intervention

3.4.1. Study Interventions Administered

Provide content from the Study Intervention(s) Administered section of the protocol and edit as applicable. The level of detail provided should account for CCI. An example table from the Common Protocol Template is provided. Cross-reference to relevant appendices should be added as needed.

Commented [BWG93]: Include this instructional text to account for disclosure considerations.

The study interventions (e.g. pharmaceuticals, devices, digital tools, or diagnostic agents) to be administered in each arm of the study, and for each period of the study, should be described, as per protocol, including route and mode of administration, dose and dosage schedule.

Where the study intervention is an add-on treatment to the current standard of care (which may be variable) then the standard of care should be described as in the protocol, but should be clearly distinguished from study intervention. Other non-investigational study interventions (such as concomitant therapy, rescue medication, challenge agents etc.) administered should be described in Section 3.4.4 and be clearly distinguished from study intervention.

For batch numbers, select the optional text that is applicable to the study. Batch numbers per participant are no longer required for most submissions but must be available if requested by health authorities.

The study intervention(s) are outlined in Table 1. The justification for the dose(s) selected is described in the justification for dose section of the protocol (Appendix [X.X.X] Study Protocol).

© 2016, 2018 TransCelerate BioPharma

<Start of suggested text>

Table 1. Study Intervention(s) Administered

**ARM Name**	Arm Name	Arm Name	Arm Name
**Intervention Name**	Intervention Name	[Placebo]	[Any additional products, including rescue medications or challenge agents]
**Type**	[Drug/Device/Biologic]	[Drug/Device/Biologic]	[Drug/Device/Biologic]
**Dose Formulation**	[tablet/ampule/capsule]	[tablet/ampule/capsule]	[tablet/ampule/capsule]
**Unit Dose Strength(s)**	[experimental, placebo,active comparator etc. from list above]	[experimental, placebo,active comparator etc. from list above]	[experimental, placebo,active comparator etc. from list above]
**Dosage Level(s)**	[dose amount and frequency]	[dose amount and frequency]	[dose amount and frequency]
**Route of Administration**	[oral/IM/IV infusion/IV injection]	[oral/IM/IV infusion/IV injection]	[oral/IM/IV infusion/IV injection]
**Use**	[experimental, placebo-active-comparator, sham comparator, rescue medication, background intervention, challenge agent, diagnostic, or other.]	[experimental, placebo-active-comparator, sham comparator, rescue medication, background intervention, challenge agent, diagnostic, or other.]	[experimental, placebo-active-comparator, sham comparator, rescue medication, background intervention, challenge agent, diagnostic, or other.]
**IMP and NIMP**	IMP and NIMP	IMP and NIMP	IMP and NIMP
**Sourcing**	[Insert/modify as appropriate: Provided centrally by the Sponsor or locally by the trial site, subsidiary, or designee. IF device list Manufacture]	[Insert/modify as appropriate: Provided centrally by the Sponsor or locally by the trial site, subsidiary, or designee. IF device list Manufacture]	[Insert/modify as appropriate: Provided centrally by the Sponsor or locally by the trial site, subsidiary, or designee. IF device list Manufacture]

Commented [BWG94]: This table is a good addition. However, it should not be mandatory because if all of the drugs have the same packaging, labeling, sourcing, route of administration etc., then it could become highly repetitious. In this case, text or bullets might be neater.

Commented [BWG95]: Suggest title case rather than all upper case is used – otherwise 'ARM' appears to be an abbreviation.

Commented [BWG96]: Should this be IMP **or** NIMP? It cannot be both

© 2016, 2018 TransCelerate BioPharma

**Packaging and Labeling**	Study Intervention will be provided in [container]. Each [container] will be labeled as required per country requirement	Study Intervention will be provided in [container]. Each [container] will be labeled as required per country requirement	Study Intervention will be provided in [container]. Each [container] will be labeled as required per country requirement
[Current/Former Name(s) or Alias(es)]	Current Names Aliases	Current Names Aliases	Current Names Aliases

[Options for batch numbers:]

[Option 1:] The manufacturing lot numbers for the study intervention(s) available to be dispensed in this study are provided [below [OR] in Appendix [X.X.X] Listing of participants receiving test drug(s)/investigational product(s) from specific batches, where more than one batch was used].

[Option 2:] A listing of batch numbers is available upon request.

[Option if the 'by-participant' listing is required:] A list of participants receiving each batch of study intervention is available in Appendix [X.X.X] Listing of participants receiving test drug(s)/investigational product(s) from specific batches, where more than one batch was used.

<End of suggested text>

3.4.2. Measures to Minimize Bias

Sample text referencing the Measures to Minimize Bias section of the protocol is provided; delete if not applicable. Provide additional subheadings and cross references to the protocol as needed to describe other measures taken (e.g., independent adjudication for endpoints or DSMB).

<Start of suggested text>

Allocation

The method used to assign/allocate participants to intervention(s) groups, including any stratification factors, if applicable, is described in in the measures to minimize bias section of the protocol (Appendix [X.X.X] Study Protocol).

Blinding

The method used for blinding/masking is described in the measures to minimize bias section of the protocol (Appendix [X.X.X] Study Protocol). [OR] This was an open-label study.

<End of suggested text>

Commented [BWG97]: Manuscript Table 1 Major Critical Review Finding: We again suggest that some detail/suggested text is included here. This only requires a brief descriptive statement, including use of IVRS, if applicable.

© 2016, 2018 TransCelerate BioPharma

3.4.3. Study intervention Compliance

Text referencing the appropriate section of the protocol is provided. Delete section if not relevant to study.

The method(s) used to assess study intervention compliance [is/are] described in the study intervention compliance section of the protocol (Appendix [X.X.X] Study Protocol).

3.4.4. Prior, Concomitant, [and/or] Post-intervention Therapy

The [medication(s)/treatment(s)/vaccination(s)/device(s)] allowed or disallowed [before, during, and/or after study intervention], including any exceptions to these requirements, are described in the Concomitant Therapy section of the protocol (Appendix [X.X.X] Study Protocol).

3.5. Study Assessments and Procedures

3.5.1. Planned Measurements and Timing of Assessments

The specific [efficacy, immunogenicity, PK, PD, safety and/or other variables to be assessed] assessments, their schedule and measurement/collection methods are provided in the Schedule of Activities and described in the Procedures sections of the protocol (Appendix [X.X.X] Study Protocol). The collection and assessment of safety information during the study (evaluation, definitions, recording, and reporting of AEs, SAEs [ADEs, SADEs] and other reportable safety events) is detailed in the AE reporting section of the protocol (Appendix [X.X.X] Study Protocol).

Commented [BWG98]: Manuscript Table 1 Major Critical Review Finding: Note that there would need to be hyperlinks taking the reader/reviewer to the exact target location within the document. For example, this hyperlink should take the reviewer to the 'Schedule of Assessments' table in the protocol rather than to the beginning of the protocol. However, from a publishing perspective, this is not so easy to achieve, and is usually only done for submission CSRs. This may mean that a reader will have to scroll through the entire protocol to find the relevant section. Also given the critical nature of AE collection, if there are special conditions governing assessment and reporting, these should be described in the CSR.

3.5.2. Appropriateness of Measures

If estimands (and/or endpoints) assessed in this study were novel or nonstandard, provide additional text identifying them and a brief description of how/why they were selected.

The [estimands and/or endpoints] used in this study (e.g., [efficacy, immunogenicity, PK, PD, safety and other endpoints], as applicable) were standard, generally reliable, and relevant to the objectives set forth in the protocol [Appendix [X.X.X] Study Protocol].

Commented [BWG99]: We suggest that the primary endpoint measure should not be regarded as only 'generally reliable' and we suggest that 'considered to be reliable' is the more appropriate wording.

3.5.3. Additional Summary of Specific Assessments

Optional section; delete if not applicable. Additional subsections may be included in this section, only if needed to describe information not available in the protocol. The protocol should be referenced whenever possible.

3.6. Data Quality Assurance

Optional subsections and suggested text are provided. Any steps taken at the investigation site or centrally to ensure the use of standard terminology and the collection of accurate, consistent, complete and reliable data, such as training sessions, monitoring of Investigators by Sponsor personnel, instruction manuals, data verification, cross-checking, use of a central laboratory for certain tests, centralized ECG reading or data audits, should be described. It should be noted whether Investigator meetings or other steps were taken to prepare Investigators and standardize performance.

The date of database release may be included.

© 2016, 2018 TransCelerate BioPharma

Any misconduct/serious GCP noncompliance information is to be documented in all relevant sections of the clinical study report (e.g., relevant subsections of this section, Section 4.2 Protocol Deviations).

If predefined quality tolerance limits (QTLs) were included in the protocol, describe in this section (subsection may be added if needed for clarity). Briefly summarize any important deviations from the predefined QTLs. If an important deviation from a QTL also qualified as an important protocol deviation, also describe it in Section 4.2 Protocol Deviations.

3.6.1. Study Monitoring

<Start of suggested text>

Study centers were monitored by [sponsor and/or partner and/or CRO]. Centers were visited at regular intervals and a Visit Log was maintained. Monitors were responsible for reviewing adherence to the protocol; compliance with GCP; and the completeness, accuracy, and consistency of the data. Direct access to subject medical and laboratory records was permitted to verify entries on the study-specific CRFs.

<End of suggested text>

3.6.2. Investigator Meetings and Staff Training

<Start of suggested text>

Investigator staff training was provided by the [define sponsor or CRO role] during [investigator meetings, initiation,] and routine monitoring visits. The Sponsor organized investigator and clinical research associate meetings before study start and during the study to provide information on the investigational product, the study rationale and design, responsibilities under [ICH/FDA/GCP], and training on the detailed study requirements.

<End of suggested text>

3.6.3. Standardization of Laboratory Procedures

Documentation of inter-laboratory standardization methods and laboratory QA procedures (e.g., laboratory validation procedures and/or certificates, equipment calibration, internal QC or external QA procedures), if used, should be provided in the appendices (e.g., Documentation of Inter-laboratory Standardization Methods and Quality Assurance Procedures if used). Laboratory manuals should not be included.

<Start of suggested text>

A central laboratory, [name of laboratory], was used to analyze the [state nature of samples] samples (Appendix [X.X.X]). Where local laboratories were used, their participation in internal and external quality control, quality assurance, and accreditation schemes was evaluated by the study monitors.

<End of suggested text>

3.6.4. Investigator Responsibilities

<Start of suggested text>

Commented [BWG100]: Manuscript Table 1 Major Critical Review Finding: Normally the quality management approach would be described here – even if just to state that SOPs were followed. We suggest that this should be added to the instructional text or as a separate subsection.

Commented [BWG101]: Additionally, we suggest to include risk measurement/mitigation measures here.

Commented [BWG102]: This implies that all verification was conducted on-site. However, with the increased use of electronic systems, much of this is now conducted remotely. Is this distinction needed?

Commented [BWG103]: Technically, GCP is addressed under ICH E6.

Commented [BWG104]: This aligns with the text in CORE Reference.

© 2016, 2018 TransCelerate BioPharma

The investigators were responsible for all data entered in the CRFs and documented their review and approval of the data by signing a form verifying the validity and completeness of the data. The investigator was responsible for appropriate retention of essential study documents.

<End of suggested text>

3.6.5. Clinical Data Management

<Start of suggested text>

Case report form data were captured (via data entry by [study center personnel or the CRO]) in [a sponsor database system or a database system owned by a CRO]. Data quality checks were applied using [manual and/or electronic] verification methods. An audit trail to support data query resolution and any modification to the data was maintained.

<End of suggested text>

3.6.6. Clinical Quality Assurance Audits

For audit purposes, if the Sponsor used an independent internal or external auditing procedure, it should be mentioned here and described in the appendices; and audit certificates from each audit, if applicable and available, may be provided in the appendices (Audit Certificates) (note it is not necessary to include audit report[s]).

<Start of suggested text>

An audit(s) of this study was included as part of the independent [Sponsor or CRO] quality assessment performed by [Sponsor or CRO/Independent Contractor]. [The audit certificate for this study is provided in Appendix [X.X.X] Audit Certificates.]

[OR]

Quality audit assessments were not performed for this study.

<End of suggested text>

3.7. Statistical Analysis

3.7.1. Statistical Analysis Plan

The intent of the templated text provided is to direct the reader to the SAP (Appendix [X.X.X] Statistical Methods) or the protocol (Appendix [X.X.X] Study Protocol, if applicable) for all information regarding planned analyses without providing further detail of the planned statistical methods in this section.

The planned analyses, [comparisons, statistical tests] and determination of sample size are described in the final version of the SAP [Appendix [X.X.X] Statistical Methods] [and/or contained in the protocol [Appendix [X.X.X] Study Protocol].

Commented [BWG105]: Spacing is larger in this paragraph compared with the rest of the document.

Commented [BWG106]: More recent electronic data capture systems link to electronic medical records and data are directly transmitted to populate fields in the eCRF. Does this need to be taken into account?

Commented [BWG107]: Manuscript Table 1 Major Critical Review Finding: This is a simplistic view of how the protocol and SAP are presented, when in reality, there may have been multiple changes to the analyses in multiple protocol amendments as well as changes made that were not documented in the SAP. In such cases it will not be straightforward for the reader access these documents and know exactly which analysis was performed. Also in some cases the protocol and SAP do not agree or the information needs to be merged - and if CSR authors do not perform this task, it makes it more difficult for the reviewer to understand the statistical methodology. This also does not support comprehension or transparency.

© 2016, 2018 TransCelerate BioPharma

3.7.2. Changes in Planned Analyses Prior to Unblinding or Database Lock

It is important that the timing of analysis changes relative to blind breaking and availability of outcome results are well characterized. In this section, describe any changes in analyses that occurred after completion of the final SAP but before unblinding of the data. Changes made after study unblinding should be described separately in Section 3.7.3 Changes Following Study Unblinding/Database Lock and Post hoc Analyses.

Select appropriate template text option provided.

[Option 1]: Changes in the planned analyses for the study that were implemented by SAP [or protocol] amendment(s) are described in Appendix [X.X.X] Statistical Methods [and/or Appendix [X.X.X] Study Protocol if applicable]. Changes made after the final SAP and before unblinding or database lock (for open label studies) are described here.

[Option 2]: All changes in the planned analyses for the study were implemented by SAP [or protocol] amendment(s), as described in Appendix [X.X.X] Statistical Methods [and/or Appendix [X.X.X] Study Protocol if applicable].

[Option 3]: There were no changes in the planned analyses for the study.

3.7.3. Changes Following Study Unblinding/Database Lock and Post-hoc Analyses

Any changes made to the planned analyses following study unblinding should be briefly described in this section. Delete section if not applicable.

Commented [BWG108]: The location of this section following discussion of the SAP is logical. The categorization of changes into 2 sections using a common delineator is good. Many clinical pharmacology studies are open-label so reference to pre- and post-unblinding does not apply in such cases. Additionally, this may become complicated should a subject need to be unblinded for safety reasons, therefore TransCelerate's suggested flexibility in the titles for Sections 3.7.2 and 3.7.3 is welcome.

Somewhere before this section should there be a section on changes to the SAP before unblinding or DB lock? If the SAP has a good 'history of change' section, a cross-reference could be sufficient.

Commented [BWG109]: Clarification is needed on how post-lock analyses could be planned, e.g. if there is a long-term follow-up phase. This is well described in CORE Reference.

© 2016, 2018 TransCelerate BioPharma

4. Study Participants

General notes for all results sections: Sections 4 and 5 should present the results from the statistical outputs with objective and balanced presentation of results. Generally, tables should be associated with text that provides an interpretation of key points, but the text should not recapitulate the data in the table. If extracting results from a larger end-text table into an in-text table, care must be taken not to omit any information that would change the interpretation of the results. Not all generated outputs included in the CSR must be referenced in the text.

Commented [BWG110]: If these are not used, then why have them? The CSR should refer to all tables and figures provided in the outputs.

If it is necessary to discuss any individual participant level information in text, consider data presentations that maintain data meaning, remain in context, and avoid references to personal protected information (PPI). Summaries should focus on the population and not on participant-level data.

Commented [BWG111]: Suggest adding this text for disclosure reasons.

<Start of suggested text>

In this clinical study report, the terms participant and subject are used interchangeably.

Commented [BWG112]: Throughout the document so far, “participant” has been used. This term should be used consistently throughout.

<End of suggested text>

4.1. Disposition of Participants

Using either a flow chart (e.g., CONSORT diagram such as that shown as suggested text) and/or an in-text table, provide a clear accounting of each participant’s disposition between enrollment into the study (ie, signing informed consent) through study completion, discontinuation (study intervention termination), or early study withdrawal (cessation of both study intervention and protocol-defined procedures). The numbers of participants who were randomized to a study intervention group and who entered and completed each phase of the study (or each week/month of the study), should be provided, as well as the reasons for all post-randomization discontinuations and withdrawals, grouped by study intervention and by major reason (e.g., AE, unsatisfactory efficacy response, failure to return, lost to follow-up). Note that for transparency, reasons for discontinuations and withdrawals should not be listed as ‘Other’.

Commented [BWG113]: “Screened” participants should be included. In some cases, this precedes informed consent. This is important to preclude screening bias, as required by regulators e.g., FDA Information Sheet - Screening Tests Prior to Study Enrollment.

Cross-check the number of discontinued participants shown in the adverse event and the discontinuation summary tables and explain any discrepancies.

Commented [BWG114]: Not all discontinuations are due to AEs, so it is valid for there to be differences.

Commented [BWG115]: We suggest using “differences” rather than “discrepancies”.

It may also be relevant to provide the number of potential participants screened for inclusion and a breakdown of the reasons for excluding potential participants during screening, if this could help clarify the appropriate patient population for eventual study intervention use.

Commented [BWG116]: This should always be included - it is not optional.

<Start of suggested text>

Figure X. Example of Participant Disposition Figure (CONsolidated Standards Of Reporting Trials [CONSORT] Diagram)

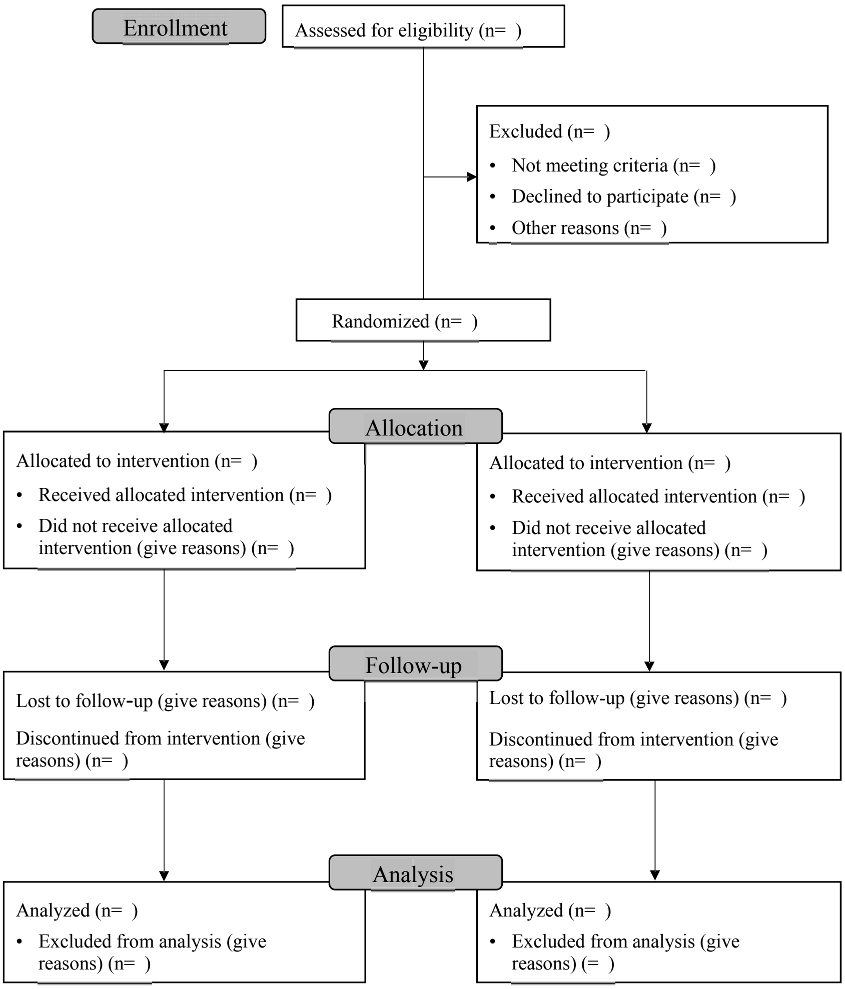

<End of suggested text>

Commented [BWG117]: We suggest that some direction should be given for handling subjects receiving the intervention that they were not randomised to. Also the analysis section of this diagram is not what is normally considered disposition so we are not sure that it should be included here.

Commented [BWG118]: Manuscript Table 1 Major Critical Review Finding:
In CORE Reference we always specify the study number in all table and figure titles. This is not done in this template which means that if the figure was copied and pasted into another document (we know that regulatory authority medical writers often do this) it would not be clear which study it was from. We suggest that the convention of adding the study number in all table and figure titles is adopted in this CSR template.

Commented [BWG119]: Manuscript Table 1 Major Critical Review Finding:
Some text in this diagram was not aligned in the boxes correctly on some computers (possibly a Mac versus PC issue). Suggest to provide (fixed and separate) file images e.g. PDFs - of any editable diagrams in the template so users can re-create their own diagrams, if necessary, using the images as a guide.

© 2016, 2018 TransCelerate BioPharma

4.2. Protocol Deviations

All important protocol deviations related to study inclusion or exclusion criteria, conduct of the study, participant management, or participant assessment should be briefly summarized, such as the following categories.

- Participants who were enrolled (as determined by the protocol) in the study even though they did not satisfy the entry criteria
- Participants who met a discontinuation or withdrawal criterion during the study but were not discontinued or withdrawn
- Participants who received the wrong study intervention or incorrect dose
- Participants who received an excluded concomitant treatment

Summaries should focus on the population and not on participant--level data.

Important protocol deviations should be listed in the appendix and presented by center for multicenter studies. Those protocol deviations that are not considered important can be referenced in the end-of-text listing.

Serious violations of GCP and/or site closures may be briefly summarized in this section with cross reference to Section 3.6 Data Quality Assurance. Add a separate section or refer to appendix (e.g., Appendix [X.X.X] Audit Certificates) for GCP noncompliance issues.

If predefined quality tolerance limits (QTLs) were included in the protocol, and if an important deviation from a QTL also qualified as an important protocol deviation, describe it here.

4.3. Populations Analyzed

A summary table, flowchart, or bulleted list of the participant evaluation groups/analysis populations is to be presented in this section.

It is assumed that all participants who received at least one dose of the study intervention are included in the safety analysis; if that is not so, an explanation should be provided. Exactly which participants were included in each analysis set should be precisely defined (e.g. all participants receiving any study intervention, all participants with any efficacy observation or with a certain minimum number of observations, only participants completing the study, all participants with an observation during a particular time window, only participants with a specified degree of compliance).

Provide a summary of participants, visits, and observations excluded from the defined (e.g., efficacy) analysis for the study, and reference the applicable appendix (e.g., Appendix [X.X.X] Participants Excluded from the [Efficacy] Analysis). Suggested text is provided.

The number of participants included in each analysis population is provided in Table X.

Commented [BWG120]: As per CORE Reference, this information would usually be either in the text or in the listing, not in an Appendix.

Commented [BWG121]: Manuscript Table 1 Major Critical Review Finding: We suggest adding a separate (sub)section for any audit findings or center-specific violations, e.g., if centers were identified with potential misconduct, identify those center IDs only here. In other parts of the CSR, cross refer to this section to avoid repeated redaction.

Commented [BWG122]: This is a good addition.

Commented [BWG123]: The template follows CORE Reference by relocating particular sections outside of what was Section 11 of ICH E3.

Commented [B124]: This should be blue text.

© 2016, 2018 TransCelerate BioPharma

<Start of suggested text>

A summary of participants excluded from the populations analyzed [by reason, timepoint] is provided in Table X; further details are provided in Appendix [X.X.X] Participants Excluded from the [Efficacy] Analysis.

<End of suggested text>

4.4. Demographic and Other Baseline Characteristics

Describe the critical demographic and baseline characteristics of the participants, as well as other factors (e.g., predefined stratification factors) arising during the study that could affect response.

This content may be sub-sectioned, for example:

4.4.1 Demography

4.4.2 Baseline Disease Characteristics

4.4.3 Medical History and Concurrent Illnesses

For studies with sites in the EU (for compliance with EU CTR), include the population of participants (including actual number of participants included in the clinical trial in the Member State concerned, in the Union, and in third [non-EU] countries); age group breakdown, gender breakdown, as applicable. Omit for non-EU studies or if no direct synopsis posting.

4.5. Prior, Concomitant, [and/or] Post-intervention Therapy

Summarize the nature and frequency of use of prior, concomitant, and post-interventional therapies, as well as possible confounding effects of the use of these therapies. Subheadings may be added for clarity.

4.6. Exposure and Study Intervention Compliance

4.6.1. Exposure

Summarize extent of exposure (dose and duration) of study intervention according to assigned group as appropriate.

4.6.2. Dose Modification

Optional Section; delete if not applicable. Summarize impact of dose modifications (dose finding/escalations, interruptions, reductions/increases, delays) to determine the degree to which safety/benefit can be assessed from the study. Reference Section 6.1.5 for dose modifications due to AEs.

4.6.3. Measurement of Compliance

Describe the degree to which participants followed their intended study intervention regimen (may be expressed as a percentage).

Commented [BWG125]: We suggest more instructional text would be useful here. Note that for disclosure, personally identifiable information, such as rare diseases of individual subjects, should not be included here.

Commented [BWG126]: In contrast to CORE Reference, this section has this as a Level 2 heading as opposed to another Level 3 heading under Demography.

Commented [BWG127]: This template includes exposure and compliance under one main heading. In CORE Reference we have compliance and exposure both as Level 2 headings.

Commented [BWG128]: CORE Reference provides guidance on how exposure can be presented - e.g. by duration, dose etc. so more instructional text would be useful here.

Commented [BWG129]: This is a good addition – and could also cover adaptive design.

Commented [BWG130]: Should be Section 5.2.1.5.

Commented [BWG131]: Manuscript Table 1 Major Critical Review Finding:
Suggest amending title to “compliance with intervention” – to distinguish it from protocol deviations (or some similar phrase that basically means “treatment compliance”).

© 2016, 2018 TransCelerate BioPharma

5. Evaluation of Response to Study Intervention

Optional sections have been provided below; delete any that are not applicable to the study.

General notes for all results sections: Sections 4 and 5 should present the results from the statistical outputs with objective and balanced presentation of results. Generally, tables should be associated with text that describes key points, but the text should not recapitulate the data in the table. If extracting results from a larger end-text table into an in-text table, care must be taken not to omit any information that would change the interpretation of the results. Not all generated outputs included in the CSR must be referenced in the text.

If it is necessary to discuss any individual participant-level information in text, consider data presentations that maintain data meaning, remain in context, AND conform to current minimum standards for de-identifying data.

Provide any results (not methods) from any statistical issues/findings/considerations (e.g., changes to the analyses, baseline imbalance, missing data, multiple comparisons) within the applicable analysis subsection within Section 5. Subheadings may be inserted as applicable for relevant topics (e.g., Post-hoc Analyses; Adjustments for Covariates; Handling of Withdrawals, Discontinuations, or Missing Data; Interim Analysis and Data Monitoring; Multicenter Studies; Use of an "Efficacy Subset" of Participants; Examination of Subgroups; or Regional Issues).

5.1. Efficacy

Delete this section if not applicable to the study.

Effectiveness is used for Medical device studies whereas for drugs, *efficacy* is used. Update header to *Effectiveness* for Medical device studies.

Subsections may be included as applicable, depending on the different analyses conducted. If estimands were implemented in the study, describe for each the applicable analyses (e.g., primary analysis, sensitivity analyses, and (optional) supplementary analyses). Subheadings could include, for example:

5.1.1 Primary Efficacy Estimand (or Endpoint)

5.1.2 Secondary Efficacy Estimand (or Endpoint)

5.1.3 Exploratory Efficacy Estimand (or Endpoint)

5.1.4 Post-hoc Analysis

Subheadings could also be based on specific endpoints and the various analyses associated with that endpoint.

If a planned analysis is to be conducted at another time point so that results are not included in this CSR (e.g. a follow-up for survival after 1 year), this should be stated.

Commented [BWG132]: We are not confident that this wording for the results section title will be readily understood or widely adopted. The issue is having this as the Level 1 results header with no mention of Efficacy or PK, as applicable. We are also not confident that "response" is applicable to safety. Is there any reason not to just title this section "Results"?

Commented [BWG133]: As per earlier comment, all generated outputs from the analyses described in the CSR should be referenced.

Commented [BWG134]: Efficacy and Safety should each be Level 1 headings and this seems a waste of the Level 1 heading. However, this structure does have the advantage of being flexible for different types of study. If splitting, need to make sure that the structure works well for earlier phase studies (e.g., phase 1 PK studies).

Commented [BWG135]: This is similar to the layout suggested by CORE Reference.

Commented [BWG136]: There is no efficacy results summary as we suggest in CORE Reference - just the summary in the Synopsis. The BWG suggest the summary of efficacy (and later on the summary of safety) are useful to consolidate everything, particularly if the Synopsis only provides the results in a table. Nowhere now apart from the overall conclusions are the efficacy and safety results consolidated and the meaning of the results made clear. Summaries can also be useful when establishing a Therapeutic Index and this will also have value in the preparation of the follow-on ISS and ISE documents, as required by FDA.

© 2016, 2018 TransCelerate BioPharma

5.2. Safety

Instructional text for all of Section 5.2 is provided in one window; please scroll down to view instructions for each subsection.

Data Transparency Tips:

- Subsections with subheadings are recommended for individual participant information (to ease redaction).
- Participant IDs (if needed) should be listed directly next to other personal information, e.g., participant 's age or sex (to ease redaction).
- Avoid using sex-specific language like "he/she, his/her," and replace with "the participant('s)." Likewise, use " participant's spouse/partner" instead of " participant's husband/wife".
- Avoid including summaries of narratives (mini-narratives) in the body of the CSR.
- Avoid use of investigator verbatim text that could include clues to the identity of the participant.
- Prefix each participant ID with '#'. This makes participant IDs easily searchable (to ease redaction).

5.2.1. Adverse Events

5.2.1.1. Brief Summary of Adverse Events

Briefly summarize the overall adverse event study results. Suggest a tabular format (e.g., insertion of the applicable summary table).

The incidence of all TEAEs reported during the study was [similar or describe differences between groups] between the study intervention groups (Table 2).

<Start of suggested text>

Commented [BWG137]: Manuscript Table 1 Major Critical Review Finding: Suggest inclusion of some tips to aid transparency (in light of the CSR being made publicly available).

Commented [BWG138]: This template just provides headings and very little guidance. CORE Reference provides many other details to help the writer. It would be very challenging for a less experienced writer to work with this template, and also the lack of guidance could lead to many different and inconsistent approaches.

© 2016, 2018 TransCelerate BioPharma

Table 2. Summary of Adverse Events

	Study Intervention [1] (N = XX) n (%)	Study Intervention [2] (N = XX) n (%)
All treatment emergent adverse events	n (%)	n (%)
Treatment-related AEs	n (%)	n (%)
Fatal AEs	n (%)	n (%)
SAEs	n (%)	n (%)
Treatment-related SAEs		
AEs leading to discontinuation of IP	n (%)	n (%)
AEs leading to discontinuation from study	n (%)	n (%)
AEs of interest	n (%)	n (%)

<End of suggested text>

5.2.1.2. Analyses of All Adverse Events

Briefly describe TEAEs by various categories as applicable, referencing supportive summary table(s). Subsections may be included as applicable, such as:

5.2.1.2.1 Frequency of AEs by Preferred Term

5.2.1.2.2 Frequency of AEs by System Organ Class

5.2.1.2.3 Frequency of AEs by Subgroups

5.2.1.2.4 Adverse Events by Severity

5.2.1.2.5 Treatment-related AEs

The presentation may be limited to selected thresholds for the study (e.g., those in at least 1% or 5% of the treated group, or other thresholds appropriate to the study).

5.2.1.3. Deaths

Briefly describe deaths occurring during the study, including the pre-treatment (Screening) period, post-intervention follow-up period and deaths that resulted from a process that began during the study. State if no events of death were reported in the study. If it is necessary to discuss any individual participant level information in text, consider data presentations that maintain data meaning, remain in context AND conform to current minimum standards for de-identifying data. Reference participant narratives in appendices as applicable.

Commented [BWG139]: This should be 'study intervention'.

Commented [BWG140]: Manuscript Table 1 Major Critical Review Finding: Inclusion of example tables would have been helpful - if not essential - for a template. The instructional text does not state that participant and events counts must be included – as we do in CORE Reference. The lack of detail in the instructional text could easily result in only participant or only event counts being presented.

Commented [BWG141]: Usually these are presented / discussed together.

Commented [BWG142]: Suggest to move subgroup analysis to below severity and relationship to treatment.

Commented [BWG143]: No Level 3 sub-heading grouping Deaths, SAEs, Discontinuations, AEs of Special Interest (AESIs) – as in ICH E3 and CORE Reference - but it does seem tidier.

Commented [BWG144]: We assume that this refers to disease progression, however, it is a strange way to phrase it.

Commented [BWG145]: No separate sub-section is provided for narratives, just a reference to where these can be found in the applicable sub-sections. However, narratives may not only be for 'Deaths'. Narratives are also possible for AESIs, discontinuations etc. so this cross-reference needs to be added to all applicable sections.

© 2016, 2018 TransCelerate BioPharma

5.2.1.4. Serious Adverse Events

Briefly describe clinically meaningful SAEs (e.g. SAEs, treatment-related SAEs), referencing supportive summary table(s). If it is necessary to discuss any individual participant--level information in text, consider data presentations that maintain data meaning, remain in context AND conform to current minimum standards for de-identifying data. State if no SAEs were reported in the study. Reference participant narratives in appendices as applicable.

5.2.1.5. Discontinuations and/or Dose Modifications Due to Adverse Events

Briefly summarize discontinuations of study intervention and/or withdrawal from the study due to AEs, referencing supportive summary table(s).

Dose modifications due to AEs (e.g. dose reduction, significant additional concomitant therapy) may also be described in this section; consider inserting a subsection for clarity.

Reference participant narratives in appendices as applicable.

5.2.1.6. Adverse Events of Special Interest

Optional section; delete if not applicable to study. Briefly describe AEs of special interest as predefined in the protocol, referencing supportive summary table(s). Subsections may be inserted for clarity. If it is necessary to discuss any individual participant level information in text, consider data presentations that maintain data meaning, remain in context AND conform to current minimum standards for de-identifying data. Reference participant narratives in appendices as applicable.

5.2.1.7. Other Significant Adverse Events

Optional section; delete if not applicable to study. Briefly describe any other significant AEs not discussed in above sections, referencing supportive summary table(s) as applicable. Subsections may be inserted for clarity. If it is necessary to discuss any individual participant level information in text, consider data presentations that maintain data meaning, remain in context AND conform to current minimum standards for de-identifying data. Reference participant narratives in appendices as applicable.

5.2.2. Clinical Laboratory Evaluation

The laboratory evaluations described will depend on the observed results, specific analyses performed, or known safety signals and should provide comparison between intervention groups, if applicable. Subsections may be included as applicable, such as:

5.2.2.1 Laboratory Values Over Time

Briefly describe laboratory values over time including topics such as:

- mean/median values, change from baseline, and those outside the laboratory normal range of values
- specific criteria used to identify clinically significant changes
- reference to appropriate summary table(s)

Commented [BWG146]: Should be '**Other** SAEs' as Deaths are also SAEs and the deaths are already discussed in the previous section.

Commented [BWG147]: Here the term "treatment" has been used rather than "intervention". This may be due to the wording in regulatory guidance documents. There is a need to seek alignment among authoritative sources to ensure that there is no conflation of terms or misunderstanding.

Commented [BWG148]: In CORE Reference we just cover discontinuations, not dose modifications. Including dose modifications could result in multiple additional AEs to cover. We suggest that inclusion of such a subsection should be results-dependent and suggest that some explanation of why it might be needed could be a useful addition.
Also, consider whether this should be "dosage" versus "dose". 'Amount' is not the same as 'frequency'.

Commented [BWG149]: Should be '**Other** AEs of Special Interest', i.e., these AEs are not included in the sub-sections above.

Commented [BWG150]: It is not apparent how Sections 5.2.1.6 and 5.2.1.7 might differ to the inexperienced author. For example, does 5.2.1.7 refer to those AEs that were not predetermined to be indicators of an effect that would be of particular safety concern in this particular disease - versus those that arise during the study that only then become significant – perhaps because of frequency or severity? This is a perfect example of why more instructional text would be highly beneficial.

Commented [BWG151]: These headings (5.2.2.1 through 5.2.2.3) have not been correctly formatted so they are not showing up in the document map.

© 2016, 2018 TransCelerate BioPharma

5.2.2.2 Summary of Changes by Participant

Briefly describe the analysis of individual participant changes by study intervention group (eg. "shift tables"). A reference to appropriate summary table(s) may be sufficient. Avoid including individual participant information here; instead, confine such information to the next section

5.2.2.3 Clinically Meaningful Laboratory Abnormalities

Briefly describe clinically meaningful changes (defined by the Sponsor) including topics such as:

- trends, relevance, and/or likely relation of laboratory abnormality to the study intervention (eg. dose/concentration, dechallenge effect)
- specific approach used to assess clinically meaningful abnormalities
- reference to appropriate summary table(s)

5.2.3. Other Safety Evaluations

Briefly describe any clinically meaningful findings and their clinical relevance, referencing supportive summary table(s). Subsections may be inserted or deleted as applicable. Provide any individual participant information in a subsection for ease of redaction.

If no other safety findings were clinically meaningful, insert a sentence such as that shown as suggested text.

<Start of suggested text>

There were no clinically meaningful findings in the [vital signs measurements, physical examination assessments], or other observations related to safety in this study. The assessments and observations were comparable across intervention groups [Table(s) X.X.X].

<End of suggested text>

5.2.3.1. Vital Signs

Briefly describe vital signs, referencing supportive summary table(s).

5.2.3.2. Electrocardiograms (ECGs)

Briefly describe ECG results (e.g., means over time, shifts, incidence of marked/clinically meaningful abnormalities), referencing supportive summary table(s).

5.2.3.3. Physical Examination Findings

Briefly describe physical findings, noting study intervention group differences and trends with increasing dose, if applicable. Reference supportive summary table(s).

Commented [BWG152]: In CORE Reference we also state that other tables (besides shift tables) and graphs can be used. These alternatives should be mentioned in the examples.

Commented [BWG153]: We suggest to include this sentence for disclosure reasons.

Commented [BWG154]: There are often narratives for lab abnormalities reported as SAEs (per E3 and CORE Reference). How would that work if "Narratives" only appear under the heading of 'Deaths" in the AE section – and not a separate (more commonly applicable) subsection? We suggest to reiterate the reference to applicable narratives in each section discussing the relevant parameter (e.g., Deaths, Labs, etc.).

Commented [BWG155]: Manuscript Table 1 Major Critical Review Finding:
There is no explanation of what these are, e.g., established scales, NCI-CTC etc. This is out of context for new authors who may not know how to report without consulting guidelines separately.

Commented [BWG156]: We suggest inclusion of this sentence for disclosure reasons.

© 2016, 2018 TransCelerate BioPharma

5.2.3.4. Safety Observations Related to [Medical Device OR Combination Product]

Briefly describe safety observations (e.g., adverse device effects [ADEs], ADEs with characteristic of an SAE [SADEs], serious adverse device effects which by its nature, incidence, severity or outcome has not been identified in the current version of the risk analysis report [USADEs], malfunctions, and remedial actions) related to medical device(s) or device/drug combination product(s) (e.g. those that involve a study intervention and a medical device such as a pre-filled syringe or auto-injector). Subsections may be added as applicable.

5.2.3.5. Other Observations Related to Safety

Optional section; delete if not applicable. Briefly describe other observations related to safety (e.g., pregnancy, infusion-related reactions, safety events related to immunogenicity [if not characterized as an AE of special interest; cross reference to Section 5.7 Immunogenicity if applicable]). If applicable to discuss safety events related to immunogenicity (e.g., neutralizing antibodies), reference participant narratives in appendices.

5.3. Pharmacokinetics

Delete this section if not applicable to the study. Subsections may be included as applicable.

Example subheadings could include:

5.3.1 Dose and Exposure

5.3.2 Drug Exposure and Safety

5.3.3 Drug Exposure and Response

5.4. Pharmacodynamics

Delete this section if not applicable to the study. Subsections may be included as applicable.

5.5. Genetics

Delete this section if not applicable to the study. Subsections may be included as applicable.

5.6. Biomarkers

Delete this section if not applicable to the study. Subsections may be included as applicable.

5.7. Immunogenicity

Delete this section if not applicable to the study. Subsections may be included as applicable.

5.8. [Health Economics] OR [Medical Resource Utilization and Health Economics]

Delete this section if not applicable to the study. Subsections may be included as applicable.

Note that this section does not apply to clinical outcome assessments (COAs). All COA parameters (e.g., patient-reported outcome measures, observer-reported outcome measures, clinician-reported outcome measures, performance outcome measures) should be fully integrated into the appropriate results sections (e.g., efficacy (Section 5.1) and/or safety (Section 5.2.3.5) sections).

Commented [BWG157]: Manuscript Table 1 Major Critical Review Finding: We would assume for pure device studies, the whole of Section 5.2 would be modified to describe the safety of the device (with section headings revised accordingly), so is this section only designed for drug studies where the drug is being delivered by a standard device? More instructional text would help the author to decide what to include. There is also a need to consider that in some jurisdictions, even when it is a drug/device combination, the two elements are reviewed by separate (e.g., drug & device) divisions.

Commented [BWG158]: There is no safety results summary as noted for the efficacy section.

Commented [BWG159]: Or should this be Section 14.3.3 (i.e. not 'appendices')?

Commented [BWG160]: Manuscript Table 1 Major Critical Review Finding: There is insufficient instructional text here. For example, there is no indication that output from PK PK/PD modeling needs to be included. Similarly, tabulation of summary PK data, presentation of summary concentration-time plots or similar should be included. If PK is the primary objective then the CSR author will need to integrate this with sub headings in Section 5.1 i.e. Section 5.1.1 primary objective: PK of X in presence of 3A4 inhibitor – for example.

Commented [BWG161]: The position of this subsection is in contrast to CORE Reference which has PK/PD/etc following efficacy but before safety. We suggest the placement for the PK section needs to be before safety, otherwise, for a clinical pharmacology study, the results of the primary endpoint would be hidden away after safety, AEs etc. We also suggest to include instructional text that states the order needs to be flexible depending on the nature of the study.

Commented [BWG162]: Instructional text is required with regard to inclusion of summary plots and tables, where appropriate. Linkage to drug exposure and response may also be required.

Commented [BWG163]: In some cases biomarkers are PD endpoints so in these cases it is inappropriate to single biomarkers out as a separate section. However, some biomarkers are not related to PD e.g. in a cognitive study, stroke, white matter lesions, lacunae, etc, may all be important biomarkers, yet have no relation to PD. Further guidance through instructional text is needed on placement of these different types of biomarker data.

Commented [BWG164]: Section 5.2.3.5 states "safety events related to immunogenicity" should be described in that section so it is not clear about the difference between these 2 sections.

Commented [BWG165]: This is a very clear and good instructional paragraph.

© 2016, 2018 TransCelerate BioPharma

5.9. [Other]

Additional subsections may be added, as necessary, to encompass other analyses not already listed above (e.g., outcomes not related to safety for medical devices, digital tools, apps, wearables). Delete section if not applicable to the study.

5.10. Summary of Evaluation of Response to Study Intervention

Optional section; section may be deleted. This section may be used to provide interpretation on the validity or limitations of the results if they are not provided in Section 6 Conclusions. Do not replicate information provided in Section 6 Conclusions here.

Commented [BWG166]: Manuscript Table 1 Major Critical Review Finding:
We assume this section is to encompass all results under Section 5 (efficacy and safety). If so, it needs to be mandatory and not optional. Would this be a series of bullet points covering efficacy and safety?

© 2016, 2018 TransCelerate BioPharma

6. Conclusions

Conclusions should be:

- presented as a bulleted list
- void of detailed interpretation about the general benefit and risk of the study intervention, which should be reserved for CTD module documents
- written simply (in plain English) and as concisely as possible
- relating back to a study objective (focusing first on primary, then secondary objectives; critical exploratory objectives could be provided if applicable)
- supported by data already presented in the CSR; do not introduce new information/data/concepts/references
- if necessary, this section may be used to explain unexpected findings and study limitations

Commented [BWG167]: Manuscript Table 1 Major Critical Review Finding:
It is exceedingly unusual to omit the Discussion section. The omission means that the reader is unable to place the results into context. The instructional text states that the risk/benefit text 'should be reserved for CTD module documents' but it is useful to have risk/benefit in the context of the individual study – as well as across studies per the CTD modules.

© 2016, 2018 TransCelerate BioPharma

7. References

- References to both internal and external documents and publications should be listed in alphabetical order. Do not reference internal reports in preparation.
- In the reference list, use the style and format published by the International Committee of Medical Journal Editors [ICMJE, 1991]. Citations to external documents and publications should be indicated in the text by citing the author and year within parentheses. For example, the in-text citation for the reference included would be (Hatcher et al, 2007).

Example of a reference:

Hatcher RA, Trussell J, Nelson AL, Cates W Jr, Stewart F, Kowal D, eds. Contraceptive technology. 19th edition. New York: Ardent Media, 2007(a): 24. Table 3-2.

End of example text

Commented [BWG168]: Often internal reports in preparation or pre-publication are referenced in the protocol. Should the CSR be in-parallel, or might there be a temporal disconnect?

Commented [BWG169]: Manuscript Table 1 Major Critical Review Finding:
No guidance is given on the tables and figures (CORE Reference/ICH E3 Section 14) or appendices (CORE Reference/ICH E3 Section 16) which seems a major omission.

© 2016, 2018 TransCelerate BioPharma
